# Development and validation of a predictive models for predicting the cardiac events within one year for patients underwent percutaneous coronary intervention procedure at IJN

**DOI:** 10.1186/s12872-023-03536-w

**Published:** 2023-11-08

**Authors:** Kok Yew Ngew, Hao Zhe Tay, Ahmad K. M. Yusof

**Affiliations:** 1 Novartis Corporation (Malaysia) Sdn Bhd, Petaling Jaya, Malaysia; 2 Department of Imaging Centre, National Heart Institute, Kuala Lumpur, Malaysia; 3 Department of Cardiology, National Heart Institute, Kuala Lumpur, Malaysia

**Keywords:** Percutaneous coronary intervention, Predictive models, Machine learning, Malaysia, Coronary artery disease

## Abstract

**Purpose:**

Percutaneous coronary intervention (PCI) is a common treatment modality for coronary artery disease. Accurate prediction of patients at risk for complications and hospital readmission after PCI could improve the overall clinical management. We aimed to develop and validate predictive models to predict any cardiac event within a year post PCI procedure.

**Methods:**

This is a retrospective cohort study utilizing data from the National Cardiovascular Disease (NCVD)-PCI registry. The data collected (N = 28,007) were split into training set (n = 24,409) and testing set (n = 3598). Four predictive models (logistic regression [LR], random forest method, support vector machine [SVM], and artificial neural network) were developed and validated. The outcome on risk prediction were compared.

**Results:**

The demographic and clinical features of patients in the training and testing cohorts were similar. Patients had mean age ± standard deviation of 58.15 ± 10.13 years at admission with a male majority (82.66%). In over half of the procedures (50.61%), patients had chronic stable angina. Within 1 year of follow up mortality, target vessel revascularization (TVR), and composite event of mortality and TVR were 3.92%, 9.48%, and 12.98% respectively. LR was the best model in predicting mortality event within 1-year post-PCI (AUC: 0.820). SVM had the highest discrimination power for both TVR event (AUC: 0.720) and composite event of mortality and TVR (AUC: 0.720).

**Conclusions:**

This study successfully identified optimal prediction models with the good discriminatory ability for mortality outcome and good discrimination ability for TVR and composite event of mortality and TVR with a simple machine learning framework.

**Supplementary Information:**

The online version contains supplementary material available at 10.1186/s12872-023-03536-w.

## Introduction

According to the World Health Organisation’s (WHO) 2019 Global Health Estimates, noncommunicable diseases make up 7 of the world’s top 10 causes of death [1]. Among these, heart disease has remained the leading cause of death at the global level for the last 20 years in which 16% of total deaths were ischaemic heart disease [2]. The number of deaths from heart disease increased steadily since 2000 rising by more than 2 million to nearly 9 million deaths in 2019 [1].

In Malaysia, cardiovascular disease (CVD) is a leading cause of morbidity and mortality. It accounted for close to 25% of total mortality and was one of the top causes of hospitalisation from a 2013 study [3]. Among all CVD conditions, coronary artery disease (CAD) accounts for the highest prevalence and mortality. Percutaneous coronary intervention (PCI) is a common treatment modality for CAD [4]. Therefore, accurate prediction of patients at risk for complications and hospital readmission after PCI could improve the overall clinical management by aiding therapy selection, enable precise preprocedural informed consent practice and reduce healthcare cost [5, 6]. There is increasing interest in developing and validating bleeding risk scores, especially in predicting whether a patient would be suitable for single or dual antiplatelet therapy post-PCI [7]. However, a few attempts at identifying risk factors for complications, mortality, and hospital readmission after PCI were met with limited success [6].

The American Heart Association/American College of Cardiology (ACC/AHA) recommended the used of risk prediction tools such as the Framingham [8], Reynolds [9], ACC/AHA [10], and QRISK2 [11] to predict future risk of CVD. However, approximately half of myocardial infarction (MI) and stroke occur in those who are not predictor to be at risk of CVD. These models also oversimplify associations by excluding a large numbers of risk factors with non-linear relationships. A better approach that incorporates multiple risk factors and determines more nuanced relationships between risk factors and outcomes need to be explored.

Continuous development in the field of computer technology has enabled the integration of medical and computational learning to create new, integrated, reliable, and efficient methods of providing quality medical care. One of the ongoing trends in cardiology at present is the utilization of machine learning (ML), a specific subset of artificial intelligence (AI), to offer an alternative approach to standard prediction modelling that may address the current limitation of these cardiac prediction assessment tools. ML can learn complex and non-linear interactions between variables [12] and has the potential to exploit various data sources for cardiac prediction algorithms development and to study pattern recognition through computational learning. In recent years, a number of studies leverage on ML to predict patient prognosis after PCI [5–6, 13–16]. However, most studies had their own limitations.

The National Cardiovascular Disease Database (NCVD) registry is a service supported by the Ministry of Health to collect information about cardiovascular disease in Malaysia to investigate the incidence of CVD, and to evaluate its risk factors and treatment in the country. The NCVD was established to integrate various CVD databases available in the country to create a nationwide cardiovascular database. NCVD maintains two different linkable databases – Acute Coronary Syndrome (ACS) database and PCI database – which has enrolled patients undergoing PCI, both elective and urgent cases in 12 centres since 2007.

This study aims to develop and validate a model to predict any cardiac event within a year post-PCI procedure, using the medical records in the NCVD-PCI database.

## Patients and methods

### Study design

This is a retrospective cohort study utilizing data from the NCVD Registry. Clinical data information was collected from the NCVD-PCI database solely focus on the single centre data available from the National Heart Institute (Institut Jantung Negara, IJN). The primary objective was to explore the performance of ML algorithms in predicting cardiac event within one year follow-up period after the initial admission of PCI. The secondary objectives were to explore the significant predictors of cardiac event occurrence by each different ML algorithms and to describe the demographic and clinical characteristics of PCI patients. The primary outcome of interest was mortality event within 1 year of followup period after discharge while the secondary outcomes of interest were the recurrence of PCI procedure which signified target vessel revascularization (TVR) and/or composite events of mortality and recurrence of PCI procedure within 1 year of followup period after discharge.

**Fig. 1:**
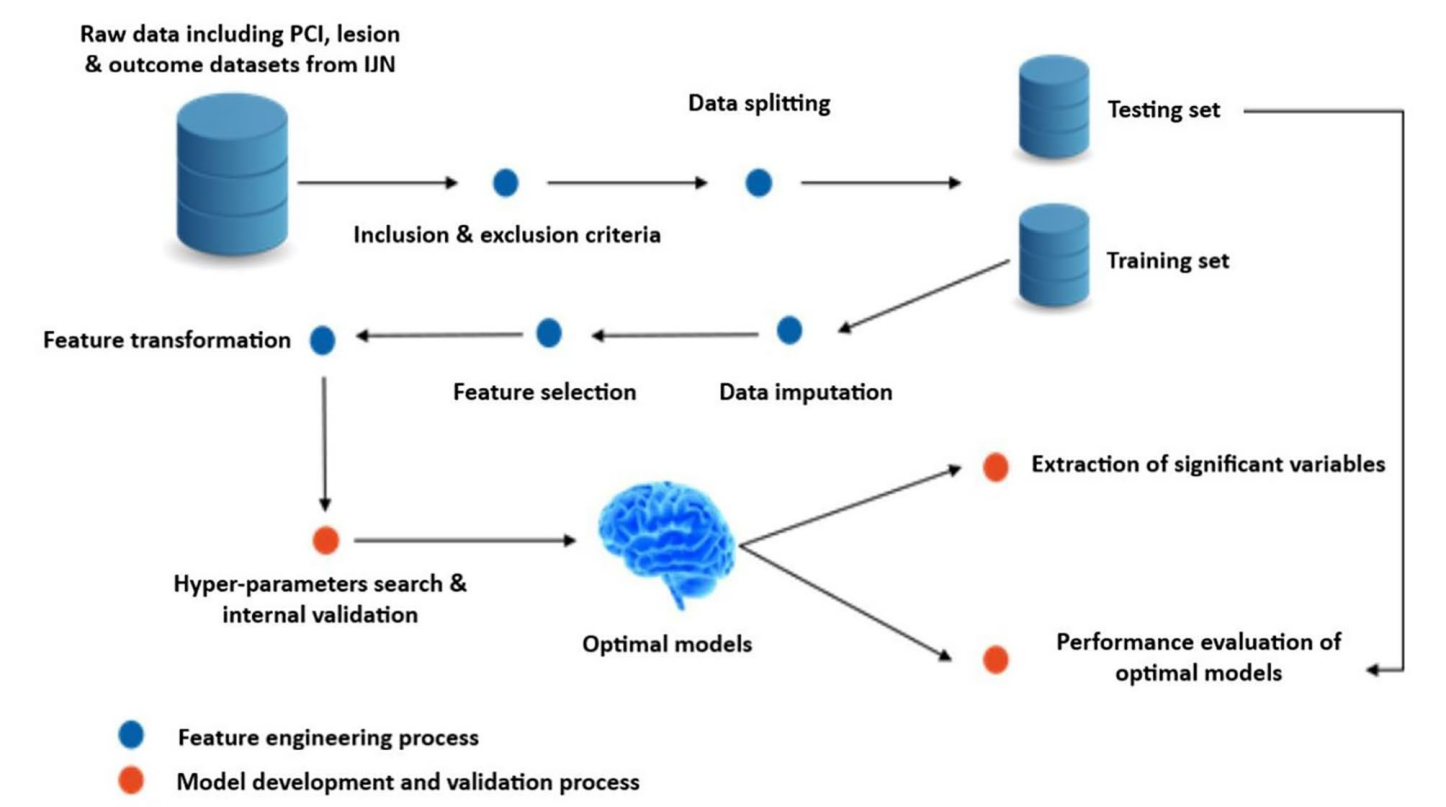
Study design and analysis flow

Four predictive models leveraging on ML were developed and validated. The outcomes on risk prediction were compared among the models (Fig. 1).

This study covered the period from 1 January 2010 to 31 December 2017 inclusive, or the latest date available in any databases at the time of data analysis. The index date was defined as the date of first PCI admission registered within the identification period. Post-index period: each patient was followed from the index date for 365 days, or until date of outcome occurrence, date of death, latest date available in both databases at the time of data collection whichever is earlier.

### Study population

#### Inclusion and exclusion criteria

All patients who underwent PCI procedure at IJN aged ≥ 18 years, between 1 January 2010 and 31 December 2016, with a complete follow-up record were included. Patients were excluded if they had > 50% incomplete information filled in the NCVD-PCI database.

### Variable information

A list of variables collected from the NCVD-PCI database is available in Supplementary Table 1. These variables including demographics, clinical status before event, clinical examination and investigation at baseline, cardiac status at PCI procedure, previous intervention, catheterization (CATH) lab visit, procedure complications, and in-hospital outcome, were used as the predictors in multivariable prediction model. All these data were systematically entered into the registry whenever a PCI procedure was performed. Follow-up data were collected at 30-day, 6-month and 12-month post-notification date intervals.

### Data analysis

#### Data splitting

The data collected from the NCVD-PCI database were split into training set and testing set. The training set covered the data from 2010 to 2015, and was used for data engineering processes, models development and internal validation. The data collected from 2016 onwards were retained as testing set for external validation purpose to estimate the performance of optimal models identified. Before fitting into the trained models to obtain prediction, the testing set would undergo the same data engineering processes as the training set.

#### Data engineering

Data engineering included data imputation, feature selection and feature transformation. Predictors recorded for less than 50% of patients in the database were not included in the model development and validation process to ensure data reliability. Any remaining missing predictor values in the database were imputed by using mode and median for categorical and continuous variables respectively.

The data were then selected by a simple filter approach using Chi-square statistics for categorical and t-test statistics for continuous variables to reduce data redundancy and improve relevancy. The top 2/3 of the variables with large absolute test statistics values were selected for further analysis.

Multi-collinearity arises when couples of predicting variables are highly related, variance inflation factor (VIF) was calculated for each variable and variables with VIF magnitude greater than 10 would then be excluded from further analysis. Categorical variables were calibrated while continuous variables were normalized to improve the utility of a feature and to ensure data is machine learnable. All remaining predictors after the entire data engineering process were fit into the ML models for investigation.

#### Model development

The following methods were used in developing the predictive models: (1) logistic regression, (2) random forest method, (3) support vector machine (SVM), and (4) artificial neural network.

Logistic regression is a statistical model that uses a sigmoid function to model a binary dependent variable. No interaction terms were considered for potential logarithm of non-linear relationships between predictors and the outcome to avoid manual specification. This was to ensure no external consideration affecting the model comparison. Regularization (or penalization) was used to overcome unstable estimates due to overfitting, collinearity, or infinite maximum likelihood estimation.

Random forest is a collection of trees predictors built by classification and regression tree (CART) methodology. In the random forest method, a pre-defined number of decision trees with limited depth of splits were trained using pre-defined training sample proportion (with replacement) and number of variables (random selection). Then, the prediction was made by taking the majority voting of all decision trees [17].

Support vector machine is a hyperplane in a high-dimensional space which was used here for classification by finding a good separation that has the maximum distance to the nearest training point [18].

Artificial neural network is a set of processing units called neurons and can be used to approximate the relationship between input and output signals of the system [19]. The hyperparameter grid search for each model is available as Supplementary Table 2.

#### Model validation

The models set were validated to avoid over-fitting and to increase the robustness of model performance. One hundred 2-folds internal validations were performed to determine the optimal hyperparameters setting for every model class. The optimal hyperparameter settings were then re-trained in the “training” and considered as best model for each method. These “best” models were then applied on “testing” set to estimate the performance. Plot of ROC curve, AUC score, accuracy, sensitivity, and specificity were reported as performance evaluation metrics. Accuracy calculations were done upon ROC construction.

#### Variable importance

The following is the variable importance extraction methodologies applied: The variable importance of logistic regression model was based on the fitted coefficient value of the model. The greater the absolute value of variable coefficient, the more important the variables. The variable importance of random forest model was calculated based on Gini importance (or known as Mean Decrease in Impurity). A variable with higher Gini importance value will have more importance in the random forest model. The variables’ effect size of SVM, was identified by the equation shown previously [18]. The variable importance of neural network was based on Olden’s algorithm [20]. All the variables were then sorted by the variable importance value calculated.

## Results

### Patient disposition and baseline characteristics

**Fig. 2:**
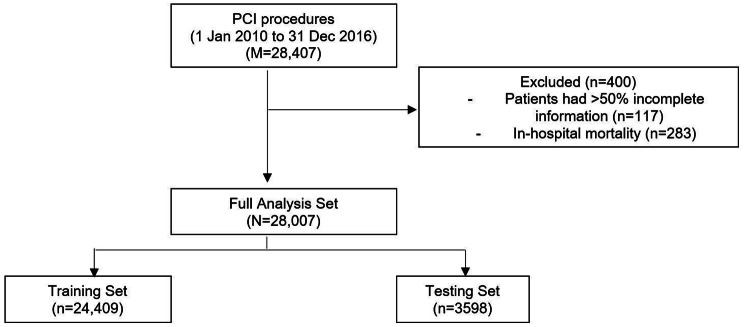
Patient disposition

Overall, 28,407 PCI procedures were performed in IJN from 1 January 2010 until 31 December 2016. 400 PCI procedures were excluded due to incomplete follow-up data (n = 117) and in-hospital mortality (n = 283), which was a competing event to the outcome of interest. In total, 28,007 procedures were included in the full analysis set (FAS) (Fig. 2). The FAS was then split into training set (n = 24,409) and testing set (n = 3598).

The demographic characteristics and cardiac status at PCI for patients underwent PCI procedures are presented in Supplementary Table 1. The demographic and clinical features of patients in the “training” and “testing” cohorts were similar. Other patient disease characteristics are available in Supplementary Table 1.

Patients had mean age ± standard deviation of 58.15 ± 10.13 years at admission with a male majority (82.66%). In over half of the procedures (50.61%), patients had chronic stable angina. Majority of the procedures skewed towards lower Canadian Cardiovascular Score (CCS) and New York Heart Association (NYHA) classification. In total, 33.03% and 45.51% of the procedures were performed on patients with CCS1 and CCS2, while 55.17% and 35.88% of the procedures were performed on patients with NYHA I and NYHA II, respectively. The majority of the patients had stable ischemic heart disease (SIHD, 74.27%) (Supplementary Table 1).

In general, the outcomes were balanced between “training” and “testing” cohorts. Majority of the procedures did not experience events of interest within 1 year of follow-up with 3.92% of mortality rate, 9.48% of TVR and 12.98% of composite event of mortality and TVR (Supplementary Table 3).

### Prediction of one year mortality

**Fig. 3:**
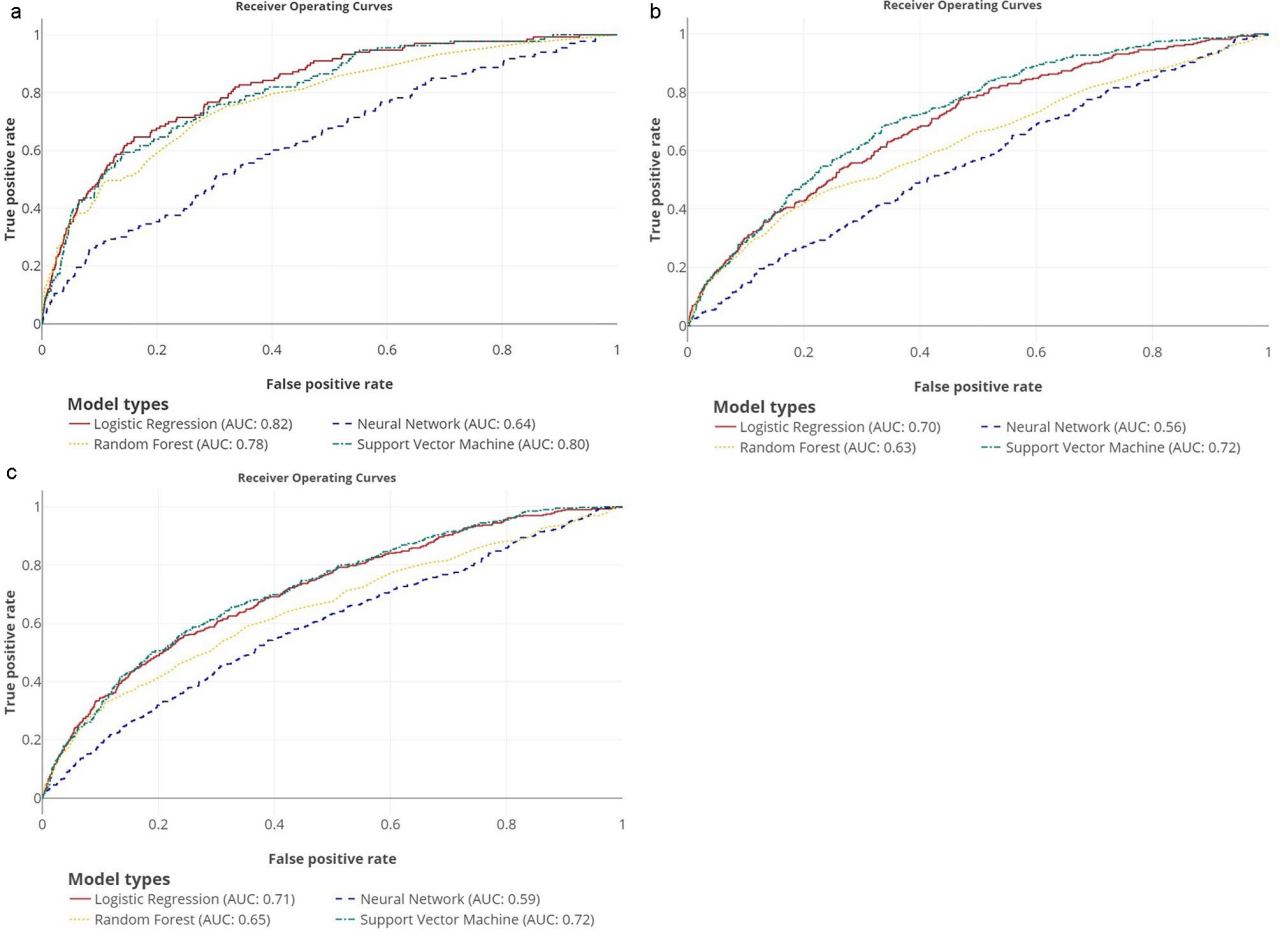
AUC for mortality events prediction. (**a**) Mortality. (**b**) TVR. (**c**) Composite events mortality and TVR AUC, area under curve; TVR, target vessel revascularization

Logistic regression was the most superior, while the neural network was the least among the models in predicting mortality event within 1-year post-PCI (Table 1). Logistic regression achieved the highest AUC score (0.820), specificity (0.840) and accuracy (0.833) and maintain a moderate sensitivity (0.647) (Table 1 and Fig. 3). The neural network scored low AUC of 0.640, sensitivity of 0.511, specificity of 0.698 and accuracy of 0.691 (Table 1 and Fig. 3).

**Table 1:** Summary of the optimal predictive models’ performance validated by testing set

	Models			
	Logistic regression	Random forest	Support vector machine	Neural network
**Mortality**				
AUC	**0.820**	0.780	0.800	0.640
Sensitivity	0.647	0.722	**0.752**	0.511
Specificity	**0.840**	0.710	0.711	0.698
Accuracy	**0.833**	0.710	0.712	0.691
**Target vessel revascularization**				
AUC	0.700	0.630	**0.720**	0.560
Sensitivity	**0.775**	0.453	0.688	0.489
Specificity	0.531	**0.771**	0.660	0.610
Accuracy	0.549	**0.746**	0.662	0.600
**Composite events of mortality and target vessel revascularization**				
AUC	0.710	0.650	**0.720**	0.590
Sensitivity	0.559	0.590	**0.656**	0.542
Specificity	**0.755**	0.648	0.674	0.611
Accuracy	**0.734**	0.641	0.672	0.604

AUC, area under curve

A total of 84 variables were identified to be predictors for mortality event (Supplementary Table 4). Age at admission, weight and acute coronary syndrome, body mass index (BMI), renal function by Cockcroft-Gault, diastolic blood pressure, heart rate at start of PCI, Modification of Diet in Renal Disease (MDRD), orally administered antihyperglycemic agents (OHA) prescription for diabetes, history of heart failure, and baseline creatinine level were potential variables that are useful in predicting the mortality event (Supplementary Table 5).

### Prediction of TVR

Support vector machine model family was the most superior while the neural network was the least in predicting TVR (Table 1). Support vector machine achieved an AUC score of 0.720 and maintain consistent performance in all metrics aspects with sensitivity of 0.688, specificity of 0.660 and accuracy of 0.662 (Table 1 and Fig. 3). The neural network had relative low ranking in all performance metrics evaluation with AUC score of 0.560, sensitivity of 0.489, specificity of 0.610 and accuracy of 0.600 (Table 1 and Fig. 3).

A total of 91 variables were identified to be predictors for TVR (Supplementary Table 4). Fluoroscopy time and pre thrombolysis in myocardial infarction (TIMI) flow were the most important variables in predicting the probability of patients’ TVR event (Supplementary Table 5). Other potential variables that are useful in determining the probability of TVR include diagnosis of SIHD, CCS, contrast volume, estimated lesion length, maximum balloon pressure, pre-PCI % of stenosis, closure device, usage of insulin, direct stenting and smoking status (Supplementary Table 5).

### Prediction of composite events of mortality and TVR

Support vector machine model family was the most superior while the neural network was the least in predicting composite events of mortality and TVR (Table 1). Support vector machine achieved AUC score of 0.720 with sensitivity of 0.656, specificity of 0.674, and accuracy of 0.672 (Table 1 and Fig. 3). The neural network has the relative low ranking in all performance metrics evaluation with AUC score of 0.590, sensitivity of 0.542, specificity of 0.611 and accuracy of 0.604 (Table 1 and Fig. 3).

A total of 90 variables were identified to be predictors for composite event (Supplementary Table 4). Estimated lesion length and fluoroscopy time are the most important variables in predicting the probability of patients’ composite event (Supplementary Table 5). Other potential variables that are useful in determining the probability of composite event include baseline creatinine level, post-PCI % of stenosis, diagnosis of SIHD, CCS, contrast volume, MDRD, usage of OHA, ethnic group, lesion type, right coronary artery (RCA) and renal function by Cockcroft Gault (Supplementary Table 5).

A full list of the predictors is available in Supplementary Table 4.

## Discussion

In this study, a total of 84, 91 and 90 variables were identified to be predictors for mortality event, TVR and composite events of mortality and TVR, respectively (Supplementary Table 4) out of a total of 184 variables available in the database after feature engineering and model development.

In this study, we identified logistic regression as the best model in predicting mortality event within 1-year post-PCI with the highest discrimination power (AUC of 0.820) while SVM had the highest discrimination power (AUC of 0.720) for both TVR event and composite event of mortality and TVR. Overall, the SVM and logistic regression model demonstrated similar and satisfactory discrimination power than a random forest and neural network model. The neural network model consistently ranked low in all predictive outcomes. Future model superiority could be determined by more advanced hyperparameters tuning such as bagging and boosting.

The current study identified SIHD of CAD to be the top significant variable in predicting mortality, TVR, and composite event. PCI procedures performed on patients with SIHD had lower mortality rate (3.64%) and TVR rate (8.98%) than those without SIHD (4.74% and 10.92%, respectively) (Supplementary Tables 6, 7). This concurs with existing literature showing that PCI in patients with acute coronary syndrome had higher mortality rate compared with those who have SIHD [21].

In line with the previous report, the 1-year mortality rate was slightly higher for NSTEMI (5.96%) than STEMI (4.39%) and unstable angina (3.34%) [22]. However, this finding seems to violate the traditional understanding that STEMI has a poorer prognosis than NSTEMI. This finding in our study may be due to patient inherent factors such as older age, co-morbidities and multivessel disease. Alkouli et al. found that the risk-adjusted rate ratio of in-hospital mortality following PCI was lower in NSTEMI as compared to STEMI [23]. It is worth noting that in-hospital mortality is usually a short-term outcome that might not be generalizable to intermediate outcome (1-year mortality). A study reported higher short-term mortality in STEMI patients but worse long-term survival after six months in non-ST-segment elevation SIHD (including NSTEMI) patients could probably explain why the 1-year mortality rate is higher in NSTEMI patients compared to STEMI patients in our analysis [24].

This study also reported that low renal function would predict 1-year post-PCI mortality. PCI procedures with 1-year mortality event reported a lower MDRD (mean value: 51.66 mL/min/1.73m^2^), lower Cockcroft-Gault (mean value: 51.24 mL/min) and higher baseline creatinine (mean value: 229.98 µmol/L) as compared to procedures without mortality (MDRD: 75.20 mL/min/1.73m^2^; Cockcroft-Gault: 78.53 mL/min; Baseline creatinine: 115.28 µmol/L). As low Cockcroft-Gault, low MDRD, and high baseline creatinine indicate of a later stage of chronic kidney disease (CKD), it is speculated that later stage CKD could be a predictor for 1-year mortality post-PCI. Increased risk of mortality after PCI for patients with end-stage CKD and low GFR was supported by previous studies [25–27].

Age at admission was identified as a significant variable for predicting of 1-year mortality events, in line with previously reported study [5, 6, 28]. A higher mean age (62.48 years) was observed for PCI procedures with mortality events than those without mortality events (57.98 years). Although the outcome measures in each study may be different, e.g., this study reported the mortality event within 1 year and Zack et al. reported mortality within 180 days, nonetheless, the positive correlation between age and mortality is evident [6].

The current study suggested a protective effect of heavier weight and higher BMI for mortality. Patients with lower mean weight (67.51 kg) and BMI (25.89 kg/m^2^) were reported to have higher procedures with mortality event than procedures without mortality (72.47 kg and 26.58 kg/m^2^, respectively). In a recent study evaluating prognostic significance of BMI after PCI in ST-elevation MI, 1-year all-cause mortality post-PCI was lower in patients with higher BMI compared with lower BMI, but such effect was non-significant after adjusting for age and other covariates [29]. A similar trend was observed in a 2006 study where obese patients had improved prognoses after PCI compared with normal-weight patients among acute MI patients [30]. It should be noted that in deriving the BMI, total body weight was used which does not differentiate between adiposity and muscle mass. Most studies using BMI as a measure also do not adjust for other prognostic variables which may vary greatly across BMI categories [31–33]. Further validation would be required to explore the different mechanisms in which anthropometric measures can contribute to beneficial effects.

Fluoroscopy time and pre TIMI flow were found to be the most common top significant variables over all the optimal models in predicting TVR events. TVR event has a higher fluoroscopy time (25.14 min) than those records without TVR event (19.87 min) (Supplementary Table 7). A positive correlation between fluoroscopy time and TVR events was also reported previously [34]. In the current study, patients with low pre TIMI level tend to have a higher TVR rate than high pre TIMI level, in line with the previous study [35].

The strength of this study was that utilisation of the NCVD-PCI database that allowed us to perform prediction over a longer period, i.e., 1-year mortality and/or TVR.

This study used simple imputations for missing values. Multiple imputations were not performed for sensitivity analysis, and hence performance of predictive models on a more robust environment was not evaluated. The “black-box” nature of some ML algorithms such as the neural networks and random forest may render the outcome challenging to interpret. Although the model validity was evaluated internally, the repetition of 2-fold cross validation showed nearly identical AUC in each validation set, indicating consistency of the data’s temporal structure and reliability of the results. This resulted in the consideration of more robust combinations of hyperparameters in the development of the final model. However, the model would also benefit from external validation to improve precision.

This study used a single-centre database which may have referral bias to IJN and may not be generalizable to the whole population. At this stage, model calibration was also not performed, but future development is in the plans for model calibration to increase reliability of the model.

This study is also inherent to limitations that are common to registry-based studies. Therefore, confounding information such as SYNTAX scoring [36] or other scoring data are not available. Major adverse coronary events data were also not available in the PCI database due to the complexity in obtaining this information.

In conclusion, this study successfully identified optimal prediction models with the good discriminatory ability for mortality outcome and good discrimination ability for TVR and composite event of mortality and TVR with a simple ML framework. These models also identified significant PCI related outcomes determinants from the large cohort of patients who underwent PCI at IJN between 2010 and 2016. Our study highlights the approach in ML prediction model for the development of a more precise and generalizable risk assessment for the decision of optimal revascularization strategies. After successful further validation and improvement, the model can help clinicians with real-time prediction of patients’ risk and patient safety, especially in the Malaysian population. In combination with other safety risk models such as SYNTAX scoring [36] and PRECISE-DAPT [7], clinicians would be better equipped to educate patients undergoing PCI on the possible risks as well as what clinicians would do to mitigate the risks. Patients or their carers can also be engaged to monitor delayed risks such as bleeding and renal impairment, and their associated treatment.

### Electronic supplementary material

Below is the link to the electronic supplementary material.

Supplementary Material 1

## Data Availability

The datasets used and/or analysed during the current study are available from the corresponding author on reasonable request.
