# Supplementary material for: Development and validation of a predictive models for predicting the cardiac events within one year for patients underwent percutaneous coronary intervention procedure at IJN
Source: BMC Cardiovasc Disord. 2023 Nov 8;23:545. doi: 10.1186/s12872-023-03536-w (PMC10634059; doi:10.1186/s12872-023-03536-w)
Supplement: Supplementary file 1 — Supplementary Material 1 [file 12872_2023_3536_MOESM1_ESM.doc]

**Supplemental Data**

Supplementary Table 1. Patient demographics and disease characteristic at index PCI procedure

|  | **Training Set** | **Testing Set** | **Total Procedure** |
| --- | --- | --- | --- |
|  | **N=24409** | **N=3598** | **N=28007** |
| Patient demographics | | | |
| Age at admission, mean (SD) | 58.14 (10.13) | 58.26 (10.20) | 58.15 (10.13) |
| Gender |  |  |  |
| Female | 4283 (17.55) | 573 (15.93) | 4856 (17.34) |
| Male | 20126 (82.45) | 3025 (84.07) | 23151 (82.66) |
| Ethnicity |  |  |  |
| Malay | 13892 (56.91) | 2107 (58.56) | 15999 (57.13) |
| Chinese | 3543 (14.52) | 550 (15.29) | 4093 (14.61) |
| Indian | 6420 (26.30) | 867 (24.10) | 7287 (26.02) |
| Other Malaysian | 432 (1.77) | 49 (1.36) | 481 (1.72) |
| Foreigner | 122 (0.50) | 25 (0.69) | 147 (0.52) |
| Cardiac status at PCI procedure |  |  |  |
| Angina type |  |  |  |
| Atypical | 2365 (9.69) | 983 (27.32) | 3348 (11.95) |
| Chronic Stable Angina | 12377 (50.71) | 1796 (49.92) | 14173 (50.61) |
| UA | 3198 (13.10) | 310 (8.62) | 3508 (12.53) |
| None | 6084 (24.93) | 488 (13.56) | 6572 (23.47) |
| Missing | 385 (1.58) | 21 (0.58) | 406 (1.45) |
| CCS |  |  |  |
| Asymptomatic | 1735 (7.11) | 575 (15.98) | 2310 (8.25) |
| CCS 1 | 7712 (31.59) | 1540 (42.80) | 9252 (33.03) |
| CCS 2 | 11566 (47.38) | 1179 (32.77) | 12745 (45.51) |
| CCS 3 | 1342 (5.50) | 253 (7.03) | 1595 (5.70) |
| CCS 4 | 450 (1.84) | 28 (0.78) | 478 (1.71) |
| Missing | 1604 (6.57) | 23 (0.64) | 1627 (5.81) |
| NYHA |  |  |  |
| NYHA I | 13350 (54.69) | 2101 (58.39) | 15451 (55.17) |
| NYHA II | 8839 (36.21) | 1209 (33.60) | 10048 (35.88) |
| NYHA III | 894 (3.66) | 245 (6.81) | 1139 (4.07) |
| NYHA IV | 163 (0.67) | 31 (0.86) | 194 (0.69) |
| Missing | 1163 (4.76) | 12 (0.33) | 1175 (4.20) |
| Coronary Artery Disease |  |  |  |
| STEMI: Anterior | 2338 (9.58) | 158 (4.39) | 2496 (8.91) |
| STEMI: Non anterior | 1536 (6.29) | 154 (4.28) | 1690 (6.03) |
| STEMI: Unknown | 212 (0.87) | 1 (0.03) | 213 (0.76) |
| NSTEMI | 1904 (7.80) | 143 (3.97) | 2047 (7.31) |
| UA | 709 (2.90) | 39 (1.08) | 748 (2.67) |
| Stable Ischemic heart disease | 17700 (72.51) | 3101 (86.19) | 20801 (74.27) |
| Missing | 10 (0.04) | 2 (0.06) | 12 (0.04) |
| Clinical examination and baseline investigation | | | |
| Height, cm, mean (SD) | n=21827 | n=3352 | n=25179 |
|  | 163.35 (8.43) | 164.10 (7.78) | 163.45 (8.35) |
| Weight, kg, mean (SD) | n=21836 | n=3358 | n=25194 |
|  | 72.06 (13.55) | 73.76 (14.06) | 72.29 (13.63) |
| BMI, kg/m^2^, mean (SD) | n=21710 | n=3344 | n=25054 |
|  | 26.97 (4.51) | 27.34 (4.66) | 27.02 (4.53) |
| Heart rate at start of PCI, bpm, mean (SD) | n=22799 | n=3483 | n=26282 |
|  | 71.19 (16.95) | 73.65 (14.41) | 71.52 (16.66) |
| Systolic blood pressure, mmHg, mean (SD) | n=22735 | n=3471 | n=26206 |
|  | 135.08 (24.67) | 136.55 (25.28) | 135.27 (24.75) |
| Diastolic blood pressure, mmHg, mean (SD) | n=22717 | n=3466 | n=26183 |
|  | 76.61 (16.47) | 76.93 (14.10) | 76.65 (16.18) |
| Baseline creatinine, mean (SD) | n=23980 | n=3541 | n=27521 |
|  | 119.31 (123.81) | 122.89 (134.77) | 119.77 (125.27) |
| Total cholesterol, mean (SD) | n=20604 | n=3010 | n=23614 |
|  | 4.36 (1.14) | 4.14 (1.14) | 4.34 (1.15) |
| LDL, mean (SD) | n=20373 | n=2959 | n=23332 |
|  | 2.47 (1.14) | 2.32 (0.98) | 2.45 (1.12) |
| Sinus rhythm | 22608 (92.62) | 3374 (93.77) | 25982 (92.77) |
| Atrial fibrillation | 279 (1.14) | 57 (1.58) | 336 (1.20) |
| Second and third atrioventricular block | 40 (0.16) | 0 (0) | 40 (0.14) |
| LBBB | 84 (0.34) | 2 (0.06) | 86 (0.31) |
| RBBB | 129 (0.53) | 5 (0.14) | 134 (0.48) |
| MDRD, mean (SD) | n=23980 | n=3541 | n=27521 |
|  | 74.30 (28.36) | 74.10 (27.33) | 74.28 (28.23) |
| Cockcroft-Gault, mean (SD) | n=21605 | n=3315 | n=24920 |
|  | 77.34 (33.45) | 78.95 (33.40) | 77.55 (33.45) |
| Non-Invasive Test |  |  |  |
| Stress exercise test | 622 (2.55) | 121 (3.36) | 743 (2.65) |
| Nuclear | 218 (0.89) | 153 (4.25) | 371 (1.32) |
| MRI | 117 (0.48) | 39 (1.08) | 156 (0.56) |
| Stress echo | 197 (0.81) | 52 (1.45) | 249 (0.89) |
| CT scan | 399 (1.63) | 132 (3.67) | 531 (1.90) |
| Functional Ischaemia |  |  |  |
| Positive | 3451 (14.14) | 27 (0.75) | 3478 (12.42) |
| Negative | 275 (1.13) | 1 (0.03) | 276 (0.99) |
| Equivocal | 133 (0.54) | 1 (0.03) | 134 (0.48) |
| Not applicable | 16958 (69.47) | 1790 (49.75) | 18748 (66.94) |
| Missing | 3592 (14.72) | 1779 (49.44) | 5371 (19.18) |
| Status before event for procedure at index date | | | |
| Smoking status |  |  |  |
| Never | 9457 (38.74) | 1728 (48.03) | 11185 (39.94) |
| Former (quit >30 days) | 6120 (25.07) | 527 (14.65) | 6647 (23.73) |
| Current (within last 30 days) | 5230 (21.43) | 994 (27.63) | 6224 (22.22) |
| Missing | 3602 (14.76) | 349 (9.70) | 3951 (14.11) |
| Dyslipidaemia | 18364 (75.23) | 1798 (49.97) | 20162 (71.99) |
| Hypertension | 18291 (74.94) | 2583 (71.79) | 20874 (74.53) |
| Diabetes |  |  |  |
| OHA | 9727 (39.85) | 1277 (35.49) | 11004 (39.29) |
| Insulin | 3230 (13.23) | 654 (18.18) | 3884 (13.87) |
| Non-pharmacology therapy diet therapy | 544 (2.23) | 97 (2.70) | 641 (2.29) |
| Unknown therapy | 563 (2.31) | 235 (6.53) | 798 (2.85) |
| Family history of premature cardiovascular disease | 3851 (15.78) | 309 (8.59) | 4160 (14.85) |
| Myocardial infarction history | 11458 (46.94) | 1773 (49.28) | 13231 (47.24) |
| Documented CAD | 14381 (58.92) | 1647 (45.78) | 16028 (57.23) |
| New onset angina <2 weeks | 7474 (30.62) | 1822 (50.64) | 9296 (33.19) |
| History of heart failure | 1000 (4.10) | 144 (4.00) | 1144 (4.08) |
| Cerebrovascular disease | 608 (2.49) | 103 (2.86) | 711 (2.54) |
| Peripheral vascular disease | 226 (0.93) | 19 (0.53) | 245 (0.87) |
| Chronic renal failure | 1648 (6.75) | 227 (6.31) | 1875 (6.69) |
| Previous intervention | | | |
| Previous PCI |  |  |  |
| < 365.25 days | 2669 (10.93) | 446 (12.40) | 3115 (11.12) |
| ≥ 365.25 days | 2953 (12.10) | 353 (9.81) | 3306 (11.80) |
| Previous CABG |  |  |  |
| < 365.25 days | 60 (0.25) | 6 (0.17) | 66 (0.24) |
| ≥ 365.25 days | 1050 (4.30) | 80 (2.22) | 1130 (4.03) |
| CATH lab characteristics | | | |
| PCI status |  |  |  |
| Elective | 22078 (90.45) | 3122 (86.77) | 25200 (89.98) |
| NSTEMI/UA | 1009 (4.13) | 178 (4.95) | 1187 (4.24) |
| STEMI | 1322 (5.42) | 298 (8.28) | 1620 (5.78) |
| Thrombolytic |  |  |  |
| <1 day | 190 (0.78) | 27 (0.75) | 217 (0.77) |
| ≥ 1 day | 349 (1.43) | 21 (0.58) | 370 (1.32) |
| Glycoprotein llb/llla Blockade |  |  |  |
| Prior | 179 (0.73) | 10 (0.28) | 189 (0.67) |
| During | 152 (0.62) | 2 (0.06) | 154 (0.55) |
| After | 40 (0.16) | 1 (0.03) | 41 (0.15) |
| Heparin | 23685 (97.03) | 3595 (99.92) | 27280 (97.40) |
| LMWH | 552 (2.26) | 116 (3.22) | 668 (2.39) |
| Ticlopidine | 598 (2.45) | 32 (0.89) | 630 (2.25) |
| Fondaparinux | 1168 (4.79) | 293 (8.14) | 1461 (5.22) |
| Bivalirudin | 6 (0.02) | 0 (0) | 6 (0.02) |
| Aspirin | 23695 (97.07) | 3508 (97.50) | 27203 (97.13) |
| Clopidogrel | 22952 (94.03) | 3069 (85.30) | 26021 (92.91) |
| 75mg | 15428 (63.21) | 2565 (71.29) | 17993 (64.24) |
| 300mg | 6520 (26.71) | 479 (13.31) | 6999 (24.99) |
| 600mg | 135 (0.55) | 4 (0.11) | 139 (0.50) |
| ≥1200mg | 0 (0) | 1 (0.03) | 1 (0.00) |
| Planned duration of clopidogrel ticlopidine |  |  |  |
| 1 month | 1081 (4.43) | 66 (1.83) | 1147 (4.10) |
| 3 months | 996 (4.08) | 47 (1.31) | 1043 (3.72) |
| 6 months | 1465 (6.00) | 48 (1.33) | 1513 (5.40) |
| 12 months | 17837 (73.08) | 2980 (82.82) | 20817 (74.33) |
| >12 months | 1382 (5.66) | 22 (0.61) | 1404 (5.01) |
| Brachial | 143 (0.59) | 2 (0.06) | 145 (0.52) |
| Femoral | 11598 (47.52) | 1341 (37.27) | 12939 (46.20) |
| Radial | 12959 (53.09) | 2625 (72.96) | 15584 (55.64) |
| Closure device |  |  |  |
| Exoseal | 211 (0.86) | 52 (1.45) | 263 (0.94) |
| Seal | 892 (3.65) | 345 (9.59) | 1237 (4.42) |
| Suture | 114 (0.47) | 28 (0.78) | 142 (0.51) |
| Others | 112 (0.46) | 2 (0.06) | 114 (0.41) |
| LAD | 6045 (24.77) | 2446 (67.98) | 8491 (30.32) |
| LCx | 3372 (13.81) | 1272 (35.35) | 4644 (16.58) |
| RCA | 4183 (17.14) | 1688 (46.91) | 5871 (20.96) |
| Graft | 512 (2.10) | 100 (2.78) | 612 (2.19) |
| LMS | 545 (2.23) | 150 (4.17) | 695 (2.48) |
| Fluoroscopy time, min, mean (SD) | n=21992 | n=3433 | n=25425 |
|  | 20.41 (24.48) | 20.13 (14.41) | 20.37 (23.38) |
| Contrast volume, min, mean (SD) | n=22462 | n=3387 | n=25849 |
|  | 186.17 (68.94) | 177.16 (61.22) | 184.99 (68.04) |
| Procedure complications | | | |
| Significant Periprocedural MI |  |  |  |
| Rise in CK/ CKMB > x3 URL | 21 (0.09) | 0 (0) | 21 (0.07) |
| Rise in Troponin > x5 URL | 25 (0.10) | 0 (0) | 25 (0.09) |
| ECG changes | 3 (0.01) | 2 (0.06) | 5 (0.02) |
| Unknown MI | 53 (0.22) | 0 (0) | 53 (0.19) |
| Emergency Reintervention/PCI |  |  |  |
| Stent thrombosis | 17 (0.07) | 3 (0.08) | 20 (0.07) |
| Dissection | 5 (0.02) | 1 (0.03) | 6 (0.02) |
| Cardiac perforation | 0 (0) | 0 (0) | 0 (0) |
| Coronary perforation | 0 (0) | 0 (0) | 0 (0) |
| New ischaemia | 5 (0.02) | 0 (0) | 5 (0.02) |
| Reinfarction | 0 (0) | 0 (0) | 0 (0) |
| Cardiac tamponade | 0 (0) | 0 (0) | 0 (0) |
| Unknown emergency | 9 (0.04) | 1 (0.03) | 10 (0.04) |
| Bail-out CABG | 0 (0) | 0 (0) | 0 (0) |
| Cardiogenic shock | 20 (0.08) | 5 (0.14) | 25 (0.09) |
| Arrhythmia (VT/VF/Brady) | 68 (0.28) | 4 (0.11) | 72 (0.26) |
| TIA/ Stroke | 11 (0.05) | 0 (0) | 11 (0.04) |
| Tamponade | 5 (0.02) | 2 (0.06) | 7 (0.02) |
| Contrast reaction | 18 (0.07) | 2 (0.06) | 20 (0.07) |
| New onset worsened heart failure | 10 (0.04) | 2 (0.06) | 12 (0.04) |
| Worsening renal impairment | 49 (0.20) | 7 (0.19) | 56 (0.20) |
| Bleeding |  |  |  |
| Minimal (Non-CNS bleeding, non-overt bleeding, <3g/dL Hb drop) | 47 (0.19) | 0 (0) | 47 (0.17) |
| Minor (Non-CNS bleeding with 3-5g/dL Hb drop) | 18 (0.07) | 0 (0) | 18 (0.06) |
| Major (Any intracranial bleed or other bleeding ≥ 5g/dL Hb drop) | 4 (0.02) | 1 (0.03) | 5 (0.02) |
| Unspecified bleeding | 2 (0.01) | 0 (0) | 2 (0.01) |
| Bleeding site |  |  |  |
| Retroperitoneal | 2 (0.01) | 0 (0) | 2 (0.01) |
| Percutaneous entry site | 49 (0.20) | 0 (0) | 49 (0.17) |
| Others | 6 (0.02) | 1 (0.03) | 7 (0.02) |
| Unspecified bleeding site | 14 (0.06) | 0 (0) | 14 (0.05) |
| Access site occlusion | 8 (0.03) | 1 (0.03) | 9 (0.03) |
| Loss of radial pulse | 0 (0) | 0 (0) | 0 (0) |
| Dissection | 22 (0.09) | 1 (0.03) | 23 (0.08) |
| Pseudo aneurysm |  |  |  |
| Ultrasound compression | 7 (0.03) | 0 (0) | 7 (0.02) |
| Surgery | 1 (0) | 0 (0) | 1 (0) |
| Others | 7 (0.03) | 3 (0.08) | 10 (0.04) |
| Unspecified pseudoaneurysm | 7 (0.03) | 0 (0) | 7 (0.02) |
| Medication prescribed post-PCI | | | |
| Aspirin | 23269 (95.33) | 3501 (97.30) | 26770 (95.58) |
| Clopidogrel | 22212 (91.00) | 2794 (77.65) | 25006 (89.28) |
| Ticlopidine | 754 (3.09) | 20 (0.56) | 774 (2.76) |
| Statin | 22916 (93.88) | 3370 (93.66) | 26286 (93.86) |
| Beta blocker | 17185 (70.40) | 2557 (71.07) | 19742 (70.49) |
| ACE Inhibitor | 12707 (52.06) | 1762 (48.97) | 14469 (51.66) |
| ARB | 3994 (16.36) | 531 (14.76) | 4525 (16.16) |
| Warfarin | 297 (1.22) | 46 (1.28) | 343 (1.22) |
| PCI Procedure details | | | |
| Coronary Lesion |  |  |  |
| De novo | 22754 (93.22) | 3338 (92.77) | 26092 (93.16) |
| Restenosis (No prior stent) | 34 (0.14) | 3 (0.08) | 37 (0.13) |
| Stent thrombosis: Acute | 25 (0.10) | 2 (0.06) | 27 (0.10) |
| Stent thrombosis: Sub acute | 20 (0.08) | 4 (0.11) | 24 (0.09) |
| Stent thrombosis: Late | 18 (0.07) | 0 (0) | 18 (0.06) |
| Stent thrombosis: Very late | 2 (0.01) | 0 (0) | 2 (0.01) |
| In stent restenosis: DES | 659 (2.70) | 78 (2.17) | 737 (2.63) |
| In stent restenosis: BMS | 339 (1.39) | 3 (0.08) | 342 (1.22) |
| Others | 51 (0.21) | 11 (0.31) | 62 (0.22) |
| Missing | 507 (2.08) | 159 (4.42) | 666 (2.38) |
| Lesion Type |  |  |  |
| A | 1121 (4.59) | 62 (1.72) | 1183 (4.22) |
| B1 | 4889 (20.03) | 959 (26.65) | 5848 (20.88) |
| B2 | 4967 (20.35) | 352 (9.78) | 5319 (18.99) |
| C | 13283 (54.42) | 2225 (61.84) | 15508 (55.37) |
| Missing | 149 (0.61) | 0 (0) | 149 (0.53) |
| LMS lesion | 320 (1.31) | 129 (3.59) | 449 (1.60) |
| Lesion Graft 18 Target Vessel* |  |  |  |
| 2 | 1 (0) | 1 (0.03) | 2 (0.01) |
| 6 | 0 (0) | 1 (0.03) | 1 (0) |
| 7 | 36 (0.15) | 5 (0.14) | 41 (0.15) |
| 8 | 18 (0.07) | 1 (0.03) | 19 (0.07) |
| 9 | 11 (0.05) | 3 (0.08) | 14 (0.05) |
| 10 | 2 (0.01) | 0 (0) | 2 (0.01) |
| 14 | 1 (0) | 0 (0) | 1 (0) |
| 16 | 1 (0) | 0 (0) | 1 (0) |
| Unspecified | 5 (0.02) | 1 (0.03) | 6 (0.02) |
| Lesion Graft 19 Target Vessel* |  |  |  |
| Unspecified | 1 (0) | 0 (0) | 1 (0) |
| Lesion Graft 20 Target Vessel* |  |  |  |
| 1 | 12 (0.05) | 8 (0.22) | 20 (0.07) |
| 2 | 9 (0.04) | 3 (0.08) | 12 (0.04) |
| 3 | 15 (0.06) | 5 (0.14) | 20 (0.07) |
| 4 | 112 (0.46) | 15 (0.42) | 127 (0.45) |
| 5 | 5 (0.02) | 0 (0) | 5 (0.02) |
| 7 | 32 (0.13) | 8 (0.22) | 40 (0.14) |
| 8 | 19 (0.08) | 2 (0.06) | 21 (0.07) |
| 9 | 6 (0.02) | 1 (0.03) | 7 (0.02) |
| 10 | 53 (0.22) | 7 (0.19) | 60 (0.21) |
| 11 | 2 (0.01) | 0 (0) | 2 (0.01) |
| 13 | 17 (0.07) | 5 (0.14) | 22 (0.08) |
| 14 | 10 (0.04) | 1 (0.03) | 11 (0.04) |
| 15 | 137 (0.56) | 30 (0.83) | 167 (0.60) |
| 16 | 21 (0.09) | 0 (0) | 21 (0.07) |
| 17 | 3 (0.01) | 0 (0) | 3 (0.01) |
| 20 | 1 (0) | 0 (0) | 1 (0) |
| Unspecified | 25 (0.10) | 2 (0.06) | 27 (0.10) |
| Lesion Graft 21 Target Vessel* |  |  |  |
| 1 | 3 (0.01) | 0 (0) | 3 (0.01) |
| 3 | 1 (0) | 0 (0) | 1 (0) |
| 4 | 11 (0.05) | 0 (0) | 11 (0.04) |
| 7 | 5 (0.02) | 0 (0) | 5 (0.02) |
| 9 | 1 (0) | 0 (0) | 1 (0) |
| 10 | 5 (0.02) | 0 (0) | 5 (0.02) |
| 13 | 1 (0) | 0 (0) | 1 (0) |
| 14 | 3 (0.01) | 0 (0) | 3 (0.01) |
| 15 | 19 (0.08) | 0 (0) | 19 (0.07) |
| 16 | 3 (0.01) | 0 (0) | 3 (0.01) |
| Unspecified | 1 (0) | 0 (0) | 1 (0) |
| Lesion Graft 22 Target Vessel |  |  |  |
| 3 | 1 (0) | 0 (0) | 1 (0) |
| 4 | 6 (0.02) | 0 (0) | 6 (0.02) |
| 7 | 1 (0) | 0 (0) | 1 (0) |
| 8 | 1 (0) | 0 (0) | 1 (0) |
| 15 | 2 (0.01) | 0 (0) | 2 (0.01) |
| 16 | 1 (0) | 0 (0) | 1 (0) |
| 17 | 1 (0) | 0 (0) | 1 (0) |
| Unspecified | 2 (0.01) | 0 (0) | 2 (0.01) |
| Lesion Graft 23 Target Vessel* |  |  |  |
| Unspecified | 2 (0.01) | 1 (0.03) | 3 (0.01) |
| Lesion Graft 24 Target Vessel* | 0 (0) | 0 (0) | 0 (0) |
| Lesion Graft 25 Target Vessel* |  |  |  |
| Unspecified | 1 (0) | 0 (0) | 1 (0) |
| Ostial | 2128 (8.72) | 386 (10.73) | 2514 (8.98) |
| CTO >3 months | 2313 (9.48) | 355 (9.87) | 2668 (9.53) |
| Calcified Lesion | 561 (2.30) | 223 (6.20) | 784 (2.80) |
| LMS | 545 (2.23) | 150 (4.17) | 695 (2.48) |
| Thrombus | 709 (2.90) | 111 (3.09) | 820 (2.93) |
| Bifurcation | 2133 (8.74) | 195 (5.42) | 2328 (8.31) |
| Pre PCI % of Stenosis, mean (SD) | n=22933 | n=2636 | n=25569 |
|  | 85.90 (18.63) | 88.74 (10.32) | 86.20 (17.98) |
| Pre PCI TIMI Flow |  |  |  |
| TIMI-0 | 3981 (16.31) | 659 (18.32) | 4640 (16.57) |
| TIMI-1 | 2337 (9.57) | 203 (5.64) | 2540 (9.07) |
| TIMI-2 | 6797 (27.85) | 699 (19.43) | 7496 (26.76) |
| TIMI-3 | 9407 (38.54) | 815 (22.65) | 10222 (36.50) |
| Missing | 1887 (7.73) | 1222 (33.96) | 3109 (11.10) |
| Post PCI % of Stenosis, mean (SD) | n=22218 | n=2926 | n=25144 |
|  | 4.03 (19.60) | 4.50 (19.72) | 4.08 (19.61) |
| Post PCI TIMI Flow |  |  |  |
| TIMI-0 | 655 (2.68) | 114 (3.17) | 769 (2.75) |
| TIMI-1 | 91 (0.37) | 6 (0.17) | 97 (0.35) |
| TIMI-2 | 230 (0.94) | 37 (1.03) | 267 (0.95) |
| TIMI-3 | 21942 (89.89) | 3280 (91.16) | 25222 (90.06) |
| Missing | 1491 (6.11) | 161 (4.47) | 1652 (5.90) |
| Estimated Lesion Length (mm), mean (SD) | n=22607 | n=3403 | n=26010 |
|  | 27.46 (16.07) | 28.03 (17.73) | 27.54 (16.30) |
| Perforation during PCI | 76 (0.31) | 5 (0.14) | 81 (0.29) |
| Lesion Result, successful | 23477 (96.18) | 3449 (95.86) | 26926 (96.14) |
| Dissection during PCI |  |  |  |
| Flow limiting | 20 (0.08) | 2 (0.06) | 22 (0.08) |
| Non flow limiting | 178 (0.73) | 49 (1.36) | 227 (0.81) |
| No reflow, n (%) |  |  |  |
| Transient | 85 (0.35) | 0 (0) | 85 (0.30) |
| Persistent | 16 (0.07) | 0 (0) | 16 (0.06) |
| Maximum balloon size (mm), mean (SD) | n=22800 | n=3444 | n=26244 |
|  | 3.12 (0.90) | 3.14 (0.52) | 3.13 (0.86) |
| Maximum balloon pressure (atm), mean (SD) | n=22407 | n=3407 | n=25814 |
|  | 16.12 (3.92) | 16.45 (4.52) | 16.16 (4.01) |
| IVUS | 1208 (4.95) | 133 (3.70) | 1341 (4.79) |
| OCT | 185 (0.76) | 58 (1.61) | 243 (0.87) |
| FFR | 196 (0.80) | 87 (2.42) | 283 (1.01) |
| Aspiration Catheter | 888 (3.64) | 217 (6.03) | 1105 (3.95) |
| POBA | 279 (1.14) | 117 (3.25) | 396 (1.41) |
| Angiojet | 1 (0) | 0 (0) | 1 (0) |
| Micro Catheter | 973 (3.99) | 415 (11.53) | 1388 (4.96) |
| Coil | 0 (0) | 0 (0) | 0 (0) |
| Rotablator | 355 (1.45) | 38 (1.06) | 393 (1.40) |
| Cutting/Scoring Balloon | 696 (2.85) | 259 (7.20) | 955 (3.41) |
| Mother-in-Child Catheter | 25 (0.10) | 8 (0.22) | 33 (0.12) |
| Embolic Protection |  |  |  |
| Filter | 70 (0.29) | 0 (0) | 70 (0.25) |
| Proximal | 1 (0) | 0 (0) | 1 (0) |
| Balloon | 4 (0.02) | 0 (0) | 4 (0.01) |
| Direct Stenting | 1468 (6.01) | 32 (0.89) | 1500 (5.36) |
| Data were presented as n (%) unless otherwise stated. *Lesion graft number and anatomosis site number is based on the Modified AHA Coronary Segment Classification used in SYNTAX scoring (Ref: Yadav M, et al. Prediction of coronary risk by SYNTAX and derived scores: synergy between percutaneous coronary intervention with taxus and cardiac surgery. J Am Coll Cardiol. 2013;62(14):1219-1230) CCS, Canadian Cardiovascular Score; NSTEMI, Non-ST-elevation myocardial infarction; NYHA, New York Heart Association functional classification; PCI, percutaneous coronary intervention; SD, standard deviation; STEMI, ST-elevation myocardial infarction; UA, unstable angina; ACE, Angiotensin Converting Enzyme; ARB, Angiotensin Receptor Blocker; BMI, Body Mass Index; CAD, Coronary Artery Disease; CABG, Coronary Artery Bypass Grafting; CK/CKMB, creatine kinase/creatine kinase myocardial band; CSS, Canadian Cardiovascular Score; CT, Computerized tomography; CTO, Chronic Total Occlusion; FFR, Fractional Flow Reserve; IVUS, Intravascular Ultrasound; ; LBBB, Left Bundle Branch Block; LCx, Left Circumflex; LDL, low-density lipoproteins; LMS, Left Main Stem; LMWH, Low-Molecular-Weight Heparin; MDRD, Modification of Diet in Renal Disease; NYHA, New York Heart Association functional classification; OCT, optical coherence tomography; OHA, Oral Hyperglycemic Agents; PCI, percutaneous coronary intervention; POBA, Plain Old Balloon Angioplasty; RCA, Right Coronary Artery; TIA, Transient Ischemic Attack; TIMI, Thrombolysis in Myocardial Infarction; URL, Upper Reference Limit; VF, Ventricular Fibrillation; VT, Ventricular Fibrillation | | | |

Supplementary Table 2. Hyper-parameter grid for the development of the prediction models

| **Model** | **Hyper-parameter** | **Rstudio package** |
| --- | --- | --- |
| Logistic regression | **Regularization techniques:**  None, Least absolute shrinkage and selection operator (Lasso), Ridge  **Lambda (for Lasso and Ridge):**  0.01, 0.05, 0.05, 0.07, 0.1 | Caret |
| Random forest | **Number of trees:**  50, 100, 150, 200  **Number of variables:**  20, 40, 60, 80  **Node sides:**  5, 15, 25 | C50 |
| Support vector machines | **Kernel functions:**  Radial basis function, polynomial, vanilla, Laplacian | Kernlab |
| Artificial neural network | **Number of hidden layers:**  1, 3, 5  **Propagation method:**  resilient backpropagation with weight backtracking (rprop+), resilient backpropagation without weight backtracking (rprop-), modified globally convergent with smallest absolute gradient (sag), modified globally convergent with smallest learning rate (slr)  **Threshold for the partial derivatives of the error function as stopping criteria:**  0.01, 0.1, 0.9 | Neuralnet |

Supplementary Table 3. Outcome summary for all procedures (FAS)

| **PCI outcome within one year after discharge** | **Training Set N=24409** | **Testing Set N=3598** | **Total Procedure N=28007** |
| --- | --- | --- | --- |
| Mortality | 964 (3.95) | 134 (3.72) | 1098 (3.92) |
| Target vessel revascularization | 2378 (9.74) | 276 (7.67) | 2654 (9.48) |
| Composite mortality and target vessel revascularization | 3239 (13.27) | 396 (11.01) | 3635 (12.98) |

Data is presented as n (%) unless otherwise stated
PCI, percutaneous coronary intervention

Supplementary Table 4. Variables identified to be included in model development process for mortality event, target vessel revascularization, and composite event of mortality and target vessel revascularization

| **Parameters** | **Mortality** | **Recurrent** | **Composite** |
| --- | --- | --- | --- |
| Chronic renal failure | Yes | Yes | Yes |
| Diabetes: OHA | Yes | No | No |
| Femoral | Yes | Yes | Yes |
| History of heart failure | Yes | No | Yes |
| NYHA | Yes | Yes | Yes |
| Statin | Yes | Yes | Yes |
| Diabetes: Non pharmacology therapy diet therapy | Yes | Yes | Yes |
| ACE Inhibitor | Yes | Yes | Yes |
| Cerebrovascular disease | Yes | No | Yes |
| Gender | Yes | Yes | No |
| Lesion Graft 20 Target Vessel | Yes | Yes | Yes |
| Peripheral vascular disease | Yes | Yes | Yes |
| Canadian Cardiovascular Score (CCS) | Yes | Yes | Yes |
| Smoking status | Yes | Yes | Yes |
| LMWH | Yes | Yes | Yes |
| Rotablator | Yes | Yes | Yes |
| Acute Coronary Syndrome | Yes | Yes | Yes |
| Closure device | Yes | Yes | No |
| Worsening renal impairment | Yes | No | Yes |
| Warfarin | Yes | Yes | Yes |
| PCI status | Yes | Yes | Yes |
| Sinus rhythm | Yes | Yes | Yes |
| Angina type | Yes | Yes | Yes |
| Stress Exercise Test | Yes | No | No |
| Graft | Yes | Yes | Yes |
| Left bundle branch block (LBBB) | Yes | Yes | Yes |
| LMS | Yes | No | Yes |
| Planned duration of clopidgrel ticlopidine | Yes | Yes | Yes |
| Pseudoaneurysm | Yes | Yes | Yes |
| Aspirin_A | Yes | Yes | Yes |
| Documented CAD | Yes | Yes | Yes |
| Calcified Lesion | Yes | Yes | Yes |
| Coronary lesion | Yes | Yes | Yes |
| Rise in CKCKMB > x3 URL | Yes | No | No |
| New on-set worsened heart failure | Yes | No | Yes |
| Contrast reaction | Yes | No | No |
| Ostial | Yes | Yes | Yes |
| Lesion Graft 21 Target Vessel | Yes | Yes | Yes |
| Functional Ischaemia | Yes | Yes | Yes |
| No Reflow | Yes | No | Yes |
| Lesion Type | Yes | Yes | Yes |
| Ethnic Group | Yes | Yes | Yes |
| Dyslipidaemia | Yes | No | Yes |
| Significant Periprocedural MI | Yes | No | No |
| Lesion Graft 18 Target Vessel | Yes | Yes | Yes |
| Family history of premature cardiovascular disease | Yes | No | Yes |
| LMS_lesion | Yes | No | No |
| Clopidogrel_A | Yes | Yes | Yes |
| RCA | Yes | Yes | Yes |
| Pre PCI TIMI Flow | Yes | Yes | Yes |
| Lesion Result | Yes | Yes | Yes |
| Cutting Scoring Balloon | Yes | No | No |
| New ischaemia | Yes | Yes | No |
| Lesion Graft 22 Target Vessel | Yes | Yes | Yes |
| Post PCI TIMI Flow | Yes | Yes | Yes |
| New on-set angina <2weeks | Yes | Yes | Yes |
| Brachial | Yes | No | No |
| TIA Stroke | Yes | No | No |
| Previous PCI | Yes | Yes | No |
| Glycoprotein Ilb/llla Blockade | Yes | Yes | Yes |
| ARB | Yes | Yes | Yes |
| Bifurcation | Yes | Yes | Yes |
| Arrhythmia (VT/VF/Brady) | Yes | No | No |
| POBA | Yes | Yes | Yes |
| Clopidogrel dose | Yes | Yes | Yes |
| Ticlopidine | Yes | No | No |
| Nationality | Yes | Yes | Yes |
| Baseline creatinine | Yes | Yes | Yes |
| Modification of Diet in Renal Disease (MDRD) | Yes | Yes | Yes |
| Cockcroft Gault | Yes | No | Yes |
| Age on admission | Yes | Yes | Yes |
| Weight | Yes | Yes | Yes |
| Heart rate at start of PCI | Yes | Yes | Yes |
| Diastolic blood pressure | Yes | No | Yes |
| BMI | Yes | Yes | Yes |
| Height | Yes | No | Yes |
| Total cholesterol | Yes | Yes | No |
| Max Balloon Pressure | Yes | Yes | Yes |
| LDL Levels | Yes | Yes | No |
| Estimated Lesion Length | Yes | Yes | Yes |
| Systolic | Yes | Yes | No |
| Post Stenosis | Yes | Yes | Yes |
| Fluoroscopy time | Yes | Yes | Yes |
| Max Balloon Size | Yes | Yes | Yes |
| CTO | No | Yes | Yes |
| Micro Catheter | No | Yes | Yes |
| Direct Stenting | No | Yes | Yes |
| Diabetes: Insulin | No | Yes | Yes |
| LCx | No | Yes | Yes |
| Aspiration Catheter | No | Yes | Yes |
| Fondaparinux | No | Yes | Yes |
| Thrombus | No | Yes | Yes |
| Previous CABG | No | Yes | Yes |
| Hypertension | No | Yes | Yes |
| Stress Exercise Test: CT Scan | No | Yes | Yes |
| FFR | No | Yes | Yes |
| Thrombolytics | No | Yes | Yes |
| Dissection lesion | No | Yes | No |
| Radial | No | Yes | Yes |
| Bleeding | No | Yes | Yes |
| Myocardial infarction history | No | Yes | No |
| IVUS | No | Yes | Yes |
| Lesion Graft 25 Target Vessel | No | Yes | No |
| Embolic Protection | No | Yes | Yes |
| Ticlopidine_A | No | Yes | Yes |
| Dissection (Procedure Complication) | No | Yes | No |
| Dissection during PCI | No | Yes | Yes |
| 2nd/3rd Atrioventricular block | No | Yes | No |
| Perforation during PCI | No | Yes | Yes |
| Mother Child | No | Yes | No |
| Contrast volume | No | Yes | Yes |
| Pre Stenosis | No | Yes | Yes |
| Auto Lesion ID | No | Yes | Yes |
| Atrial Fibrillation | No | No | Yes |

ACE, Angiotensin Converting Enzyme; ARB, Angiotensin Receptor Blocker; BMI, Body Mass Index; CAD, Coronary Artery Disease; CABG, Coronary Artery Bypass Grafting; CK/CKMB, creatine kinase/creatine kinase myocardial band; CSS, Canadian Cardiovascular Score; CT, Computerized tomography; CTO, Chronic Total Occlusion; FFR, Fractional Flow Reserve; IVUS, Intravascular Ultrasound; ; LBBB, Left Bundle Branch Block; LCx, Left Circumflex; LDL, low-density lipoproteins; LMS, Left Main Stem; LMWH, Low-Molecular-Weight Heparin; MDRD, Modification of Diet in Renal Disease; NYHA, New York Heart Association functional classification; OHA, Oral Hyperglycaemic Agents; PCI, percutaneous coronary intervention; POBA, Plain Old Balloon Angioplasty; RCA, Right Coronary Artery; TIA, Transient Ischemic Attack; TIMI, Thrombolysis in Myocardial Infarction; URL, Upper Reference Limit; VF, Ventricular Fibrillation; VT, Ventricular Fibrillation

Supplementary Table 5. Top variables identified in predicting the probability of patients’ composite event mortality event, target vessel revascularization, and composite event of mortality and target vessel revascularization

| **Parameters** | **Mortality** | **TVR** | **Composite mortality and TVR** |
| --- | --- | --- | --- |
| Age on admission | Yes |  |  |
| Weight | Yes |  |  |
| Stable Ischemic Heart Disease | Yes | Yes | Yes |
| BMI | Yes |  |  |
| Renal function by Cockcroft Gault | Yes |  | Yes |
| Diastolic blood pressure | Yes |  |  |
| Heart rate at start of PCI | Yes |  |  |
| Renal function by MDRD | Yes |  | Yes |
| Diabetes: OHA | Yes |  | Yes |
| History of heart failure | Yes |  |  |
| Baseline creatinine | Yes |  | Yes |
| Fluoroscopy time |  | Yes | Yes |
| Pre PCI TIMI flow |  | Yes |  |
| Canadian Cardiovascular Score |  | Yes | Yes |
| Contrast volume |  | Yes | Yes |
| Estimated lesion length |  | Yes | Yes |
| Max balloon pressure |  | Yes |  |
| Pre PCI % of stenosis |  | Yes | Yes |
| Closure device |  | Yes |  |
| Diabetes: Insulin |  | Yes |  |
| Direct stenting |  | Yes |  |
| Smoking status |  | Yes |  |
| Ethnic group |  |  | Yes |
| Lesion type |  |  | Yes |
| RCA |  |  | Yes |

BMI, body mass index; MDRD, modification of diet in renal disease; OHA, oral antihyperglycemic agents; PCI, percutaneous coronary intervention; RCA, right coronary artery; TIMI, thrombolysis in myocardial infarction; TVR, target vessel revascularization

Supplementary Table 6. Patient demographics and disease characteristic by mortality event

|  | **Mortality event** | |  |  |
| --- | --- | --- | --- | --- |
|  | **Death** | **Alive** |  |  |
|  | **N=1098** | **N=26909** | **p-values** | **Mortality rate** |
| Patient demographics | | | | |
| Age at admission, mean (SD) | 62.48 (10.32) | 57.98 (10.09) | <0.001 |  |
| Gender |  |  |  |  |
| Female | 271 (24.68) | 4585 (17.04) | <0.001 | 5.58 % |
| Male | 827 (75.32) | 22324 (82.96) |  | 3.57 % |
| Ethnicity |  |  |  |  |
| Malay | 662 (60.29) | 15337 (57.00) | 0.066 | 4.14 % |
| Chinese | 142 (12.93) | 3951 (14.68) |  | 3.47 % |
| Indian | 274 (24.95) | 7013 (26.06) |  | 3.76 % |
| Other Malaysian | 19 (1.73) | 462 (1.72) |  | 3.95 % |
| Foreigner | 1 (0.09) | 146 (0.54) |  | 0.68 % |
| Cardiac status at PCI procedure |  |  |  |  |
| Angina type |  |  |  |  |
| Atypical | 120 (10.93) | 3228 (12.00) | <0.001 | 3.58 % |
| Chronic Stable Angina | 544 (49.54) | 13629 (50.65) |  | 3.84 % |
| UA | 188 (17.12) | 3320 (12.34) |  | 5.36 % |
| None | 224 (20.40) | 6348 (23.59) |  | 3.41 % |
| Missing | 22 (2.00) | 384 (1.43) |  | 5.42 % |
| CCS |  |  |  |  |
| Asymptomatic | 75 (6.83) | 2235 (8.31) | <0.001 | 3.25 % |
| CCS 1 | 305 (27.78) | 8947 (33.25) |  | 3.3 % |
| CCS 2 | 526 (47.91) | 12219 (45.41) |  | 4.13 % |
| CCS 3 | 97 (8.83) | 1498 (5.57) |  | 6.08 % |
| CCS 4 | 33 (3.01) | 445 (1.65) |  | 6.9 % |
| Missing | 62 (5.65) | 1565 (5.82) |  | 3.81 % |
| NYHA |  |  |  |  |
| NYHA I | 492 (44.81) | 14959 (55.59) | <0.001 | 3.18 % |
| NYHA II | 443 (40.35) | 9605 (35.69) |  | 4.41 % |
| NYHA III | 92 (8.38) | 1047 (3.89) |  | 8.08 % |
| NYHA IV | 24 (2.19) | 170 (0.63) |  | 12.37 % |
| Missing | 47 (4.28) | 1128 (4.19) |  | 4 % |
| Coronary Artery Disease |  |  |  |  |
| STEMI: Anterior | 107 (9.74) | 2389 (8.88) | <0.001 | 4.29 % |
| STEMI: Non anterior | 74 (6.74) | 1616 (6.01) |  | 4.38 % |
| STEMI: Unknown | 12 (1.09) | 201 (0.75) |  | 5.63 % |
| NSTEMI | 122 (11.11) | 1925 (7.15) |  | 5.96 % |
| UA | 25 (2.28) | 723 (2.69) |  | 3.34 % |
| Stable Ischemic heart disease | 757 (68.94) | 20044 (74.49) |  | 3.64 % |
| Missing | 1 (0.09) | 11 (0.04) |  | 8.33 % |
| Clinical examination and baseline investigation | | | | |
| Height, cm, mean (SD) | n=901 | n=24278 | <0.001 |  |
|  | 161.31 (9.39) | 163.52 (8.30) |  |  |
| Weight, kg, mean (SD) | n=902 | n=24292 | <0.001 |  |
|  | 67.51 (13.60) | 72.47 (13.60) |  |  |
| BMI, kg/m^2^, mean (SD) | n=894 | n=24160 | <0.001 |  |
|  | 25.89 (4.78) | 27.06 (4.52) |  |  |
| Heart rate at start of PCI, bpm, mean (SD) | n=1020 | n=25262 | <0.001 |  |
|  | 75.82 (17.14) | 71.34 (16.61) |  |  |
| Systolic blood pressure, mmHg, mean (SD) | n=1023 | n=25183 | 0.045 |  |
|  | 136.79 (28.82) | 135.21 (24.57) |  |  |
| Diastolic blood pressure, mmHg, mean (SD) | n=1022 | n=25161 | <0.001 |  |
|  | 72.79 (14.38) | 76.81 (16.23) |  |  |
| Baseline creatinine, mean (SD) | n=1079 | n=26442 | <0.001 |  |
|  | 229.98 (234.09) | 115.28 (116.55) |  |  |
| Total cholesterol, mean (SD) | n=859 | n=22755 | <0.001 |  |
|  | 4.18 (1.17) | 4.34 (1.14) |  |  |
| LDL, mean (SD) | n=854 | n=22478 | 0.063 |  |
|  | 2.38 (1.01) | 2.46 (1.12) |  |  |
| Sinus rhythm | 980 (89.25) | 25002 (92.91) | <0.001 | 3.77 % |
| Atrial fibrillation | 32 (2.91) | 304 (1.13) | <0.001 | 9.52 % |
| Second and third atrioventricular block | 2 (0.18) | 38 (0.14) | 0.67 | 5 % |
| LBBB | 11 (1.00) | 75 (0.28) | 0.001 | 12.79 % |
| RBBB | 6 (0.55) | 128 (0.48) | 0.912 | 4.48 % |
| MDRD, mean (SD) | n=1079 | n=26442 | <0.001 |  |
|  | 51.66 (33.14) | 75.20 (27.62) |  |  |
| Cockcroft-Gault, mean (SD) | n=894 | n=24026 | <0.001 |  |
|  | 51.24 (32.77) | 78.53 (33.07) |  |  |
| Non-Invasive Test |  |  |  |  |
| Stress exercise test | 4 (0.36) | 739 (2.75) | <0.001 | 0.54 % |
| Nuclear | 25 (2.28) | 346 (1.29) | 0.007 | 6.74 % |
| MRI | 8 (0.73) | 148 (0.55) | 0.347 | 5.13 % |
| Stress echo | 6 (0.55) | 243 (0.90) | 0.255 | 2.41 % |
| CT scan | 18 (1.64) | 513 (1.91) | 0.434 | 3.39 % |
| Functional Ischaemia |  |  |  |  |
| Positive | 109 (9.93) | 3369 (12.52) | 0.048 | 3.13 % |
| Negative | 7 (0.64) | 269 (1.00) |  | 2.54 % |
| Equivocal | 2 (0.18) | 132 (0.49) |  | 1.49 % |
| Not applicable | 732 (66.67) | 18016 (66.95) |  | 3.9 % |
| Missing | 248 (22.59) | 5123 (19.04) |  | 4.62 % |
| Status before event for procedure at index date | | | | |
| Smoking status |  |  |  |  |
| Never | 481 (43.81) | 10704 (39.78) | <0.001 | 4.3 % |
| Former (quit >30 days) | 243 (22.13) | 6404 (23.80) |  | 3.66 % |
| Current (within last 30 days) | 168 (15.30) | 6056 (22.51) |  | 2.7 % |
| Missing | 206 (18.76) | 3745 (13.92) |  | 5.21 % |
| Dyslipidaemia | 756 (68.85) | 19406 (72.12) | 0.017 | 3.75 % |
| Hypertension | 944 (85.97) | 19930 (74.06) | <0.001 | 4.52 % |
| Diabetes |  |  |  |  |
| OHA | 429 (39.07) | 10575 (39.30) |  | 3.9 % |
| Insulin | 353 (32.15) | 3531 (13.12) |  | 9.09 % |
| Non-pharmacology therapy diet therapy | 34 (3.10) | 607 (2.26) |  | 5.3 % |
| Unknown therapy | 57 (5.19) | 741 (2.75) |  | 7.14 % |
| Family history of premature cardiovascular disease | 132 (12.02) | 4028 (14.97) | 0.003 | 3.17 % |
| Myocardial infarction history | 549 (50.00) | 12682 (47.13) | 0.055 | 4.15 % |
| Documented CAD | 700 (63.75) | 15328 (56.96) | <0.001 | 4.37 % |
| New onset angina <2 weeks | 395 (35.97) | 8901 (33.08) | 0.051 | 4.25 % |
| History of heart failure | 145 (13.21) | 999 (3.71) | <0.001 | 12.67 % |
| Cerebrovascular disease | 70 (6.38) | 641 (2.38) | <0.001 | 9.85 % |
| Peripheral vascular disease | 30 (2.73) | 215 (0.80) | <0.001 | 12.24 % |
| Chronic renal failure | 332 (30.24) | 1543 (5.73) | <0.001 | 17.71 % |
| Previous intervention | | | | |
| Previous PCI |  |  |  |  |
| < 365.25 days | 133 (12.11) | 2982 (11.08) | 0.052 | 4.27 % |
| ≥ 365.25 days | 148 (13.48) | 3158 (11.74) |  | 4.48 % |
| Previous CABG |  |  |  |  |
| < 365.25 days | 4 (0.36) | 62 (0.23) | <0.001 | 6.06 % |
| ≥ 365.25 days | 72 (6.56) | 1058 (3.93) |  | 6.37 % |
| CATH lab characteristics | | | | |
| PCI status |  |  |  |  |
| Elective | 939 (85.52) | 24261 (90.16) | <0.001 | 3.73 % |
| NSTEMI/UA | 70 (6.38) | 1117 (4.15) |  | 5.9 % |
| STEMI | 89 (8.11) | 1531 (5.69) |  | 5.49 % |
| Thrombolytic |  |  |  |  |
| <1 day | 8 (0.73) | 209 (0.78) | 0.976 | 3.69 % |
| ≥ 1 day | 14 (1.28) | 356 (1.32) |  | 3.78 % |
| Glycoprotein llb/llla Blockade |  |  |  |  |
| Prior | 9 (0.82) | 180 (0.67) | 0.574 | 4.76 % |
| During | 9 (0.82) | 145 (0.54) |  | 5.84 % |
| After | 2 (0.18) | 39 (0.14) |  | 4.88 % |
| Heparin | 1070 (97.45) | 26210 (97.40) | >0.999 | 3.92 % |
| LMWH | 64 (5.83) | 604 (2.24) | <0.001 | 9.58 % |
| Ticlopidine | 30 (2.73) | 600 (2.23) | 0.319 | 4.76 % |
| Fondaparinux | 65 (5.92) | 1396 (5.19) | 0.294 | 4.45 % |
| Bivalirudin | 0 (0) | 6 (0.02) | >0.999 | 0 % |
| Aspirin | 1067 (97.18) | 26136 (97.13) | >0.999 | 3.92 % |
| Clopidogrel | 1011 (92.08) | 25010 (92.94) | 0.3 | 3.89 % |
| 75mg | 705 (64.21) | 17288 (64.25) | 0.854 | 3.92 % |
| 300mg | 268 (24.41) | 6731 (25.01) |  | 3.83 % |
| 600mg | 5 (0.46) | 134 (0.50) |  | 3.6 % |
| ≥1200mg | 0 (0) | 1 (0) |  | 0 % |
| Planned duration of clopidogrel ticlopidine |  |  |  |  |
| 1 month | 71 (6.47) | 1076 (4.00) | 0.001 | 6.19 % |
| 3 months | 41 (3.73) | 1002 (3.72) |  | 3.93 % |
| 6 months | 59 (5.37) | 1454 (5.40) |  | 3.9 % |
| 12 months | 771 (70.22) | 20046 (74.50) |  | 3.7 % |
| >12 months | 57 (5.19) | 1347 (5.01) |  | 4.06 % |
| Brachial | 10 (0.91) | 135 (0.50) | 0.106 | 6.9 % |
| Femoral | 764 (69.58) | 12175 (45.25) | <0.001 | 5.9 % |
| Radial | 345 (31.42) | 15239 (56.63) | <0.001 | 2.21 % |
| Closure device |  |  |  |  |
| Exoseal | 15 (1.37) | 248 (0.92) | <0.001 | 5.7 % |
| Seal | 75 (6.83) | 1162 (4.32) |  | 6.06 % |
| Suture | 10 (0.91) | 132 (0.49) |  | 7.04 % |
| Others | 3 (0.27) | 111 (0.41) |  | 2.63 % |
| LAD | 338 (30.78) | 8153 (30.30) | 0.757 | 3.98 % |
| LCx | 199 (18.12) | 4445 (16.52) | 0.174 | 4.29 % |
| RCA | 270 (24.59) | 5601 (20.81) | 0.003 | 4.6 % |
| Graft | 50 (4.55) | 562 (2.09) | <0.001 | 8.17 % |
| LMS | 54 (4.92) | 641 (2.38) | <0.001 | 7.77 % |
| Fluoroscopy time, min, mean (SD) | n=1002 | n=24423 | 0.149 |  |
|  | 21.42 (16.19) | 20.33 (23.63) |  |  |
| Contrast volume, min, mean (SD) | n=1004 | n=24845 | 0.874 |  |
|  | 184.66 (69.72) | 185.01 (67.98) |  |  |
| Procedure complications | | | | |
| Significant Periprocedural MI |  |  |  |  |
| Rise in CK/ CKMB > x3 URL | 4 (0.36) | 17 (0.06) |  | 19.05 % |
| Rise in Troponin > x5 URL | 4 (0.36) | 21 (0.08) |  | 16 % |
| ECG changes | 0 (0) | 5 (0.02) |  | 0 % |
| Unknown MI | 2 (0.18) | 51 (0.19) |  | 3.77 % |
| Emergency Reintervention/PCI |  |  |  |  |
| Stent thrombosis | 0 (0) | 20 (0.07) |  | 0 % |
| Dissection | 0 (0) | 6 (0.02) |  | 0 % |
| Cardiac perforation | 0 (0) | 0 (0) |  |  |
| Coronary perforation | 0 (0) | 0 (0) |  |  |
| New ischaemia | 1 (0.09) | 4 (0.01) |  | 20 % |
| Reinfarction | 0 (0) | 0 (0) |  |  |
| Cardiac tamponade | 0 (0) | 0 (0) |  |  |
| Unknown emergency | 1 (0.09) | 9 (0.03) |  | 10 % |
| Bail-out CABG | 0 (0) | 0 (0) | NE |  |
| Cardiogenic shock | 0 (0) | 25 (0.09) | 0.624 | 0 % |
| Arrhythmia (VT/VF/Brady) | 5 (0.46) | 67 (0.25) | 0.208 | 6.94 % |
| TIA/ Stroke | 2 (0.18) | 9 (0.03) | 0.067 | 18.18 % |
| Tamponade | 1 (0.09) | 6 (0.02) | 0.244 | 14.29 % |
| Contrast reaction | 5 (0.46) | 15 (0.06) | 0.001 | 25 % |
| New onset worsened heart failure | 4 (0.36) | 8 (0.03) | 0.001 | 33.33 % |
| Worsening renal impairment | 11 (1.00) | 45 (0.17) | <0.001 | 19.64 % |
| Bleeding |  |  |  |  |
| Minimal (Non-CNS bleeding, non-overt bleeding, <3g/dL Hb drop) | 1 (0.09) | 46 (0.17) | 0.349 | 2.13 % |
| Minor (Non-CNS bleeding with 3-5g/dL Hb drop) | 0 (0) | 18 (0.07) |  | 0 % |
| Major (Any intracranial bleed or other bleeding ≥ 5g/dL Hb drop) | 1 (0.09) | 4 (0.01) |  | 20 % |
| Unspecified bleeding | 0 (0) | 2 (0.01) |  | 0 % |
| Bleeding site |  |  |  |  |
| Retroperitoneal | 0 (0) | 2 (0.01) | 0.484 | 0 % |
| Percutaneous entry site | 1 (0.09) | 48 (0.18) |  | 2.04 % |
| Others | 1 (0.09) | 6 (0.02) |  | 14.29 % |
| Unspecified bleeding site | 0 (0) | 14 (0.05) |  | 0 % |
| Access site occlusion | 1 (0.09) | 8 (0.03) | 0.302 | 11.11 % |
| Loss of radial pulse | 0 (0) | 0 (0) | NE |  |
| Dissection | 2 (0.18) | 21 (0.08) | 0.227 | 8.7 % |
| Pseudo aneurysm |  |  |  |  |
| Ultrasound compression | 2 (0.18) | 5 (0.02) | 0.001 | 28.57 % |
| Surgery | 0 (0) | 1 (0) |  | 0 % |
| Others | 3 (0.27) | 7 (0.03) |  | 30 % |
| Unspecified pseudoaneurysm | 1 (0.09) | 6 (0.02) |  | 14.29 % |
| Medication prescribed post-PCI | | | | |
| Aspirin | 1023 (93.17) | 25747 (95.68) | <0.001 | 3.82 % |
| Clopidogrel | 967 (88.07) | 24039 (89.33) | 0.252 | 3.87 % |
| Ticlopidine | 33 (3.01) | 741 (2.75) | 0.682 | 4.26 % |
| Statin | 963 (87.70) | 25323 (94.11) | <0.001 | 3.66 % |
| Beta blocker | 773 (70.40) | 18969 (70.49) | 0.987 | 3.92 % |
| ACE Inhibitor | 434 (39.53) | 14035 (52.16) | <0.001 | 3 % |
| ARB | 159 (14.48) | 4366 (16.23) | 0.138 | 3.51 % |
| Warfarin | 32 (2.91) | 311 (1.16) | <0.001 | 9.33 % |
| PCI Procedure details | | | | |
| Coronary Lesion |  |  |  |  |
| De novo | 1004 (91.44) | 25088 (93.23) | 0.087 | 3.85 % |
| Restenosis (No prior stent) | 2 (0.18) | 35 (0.13) |  | 5.41 % |
| Stent thrombosis: Acute | 0 (0) | 27 (0.10) |  | 0 % |
| Stent thrombosis: Sub acute | 3 (0.27) | 21 (0.08) |  | 12.5 % |
| Stent thrombosis: Late | 0 (0) | 18 (0.07) |  | 0 % |
| Stent thrombosis: Very late | 0 (0) | 2 (0.01) |  | 0 % |
| In stent restenosis: DES | 42 (3.83) | 695 (2.58) |  | 5.7 % |
| In stent restenosis: BMS | 14 (1.28) | 328 (1.22) |  | 4.09 % |
| Others | 1 (0.09) | 61 (0.23) |  | 1.61 % |
| Missing | 32 (2.91) | 634 (2.36) |  | 4.8 % |
| Lesion Type |  |  |  |  |
| A | 41 (3.73) | 1142 (4.24) | 0.020 | 3.47 % |
| B1 | 195 (17.76) | 5653 (21.01) |  | 3.33 % |
| B2 | 202 (18.40) | 5117 (19.02) |  | 3.8 % |
| C | 654 (59.56) | 14854 (55.20) |  | 4.22 % |
| Missing | 6 (0.55) | 143 (0.53) |  | 4.03 % |
| LMS lesion | 32 (2.91) | 417 (1.55) | 0.001 | 7.13 % |
| Lesion Graft 18 Target Vessel* |  |  |  |  |
| 2 | 0 (0) | 2 (0.01) | 0.431 | 0 % |
| 6 | 0 (0) | 1 (0) |  | 0 % |
| 7 | 1 (0.09) | 40 (0.15) |  | 2.44 % |
| 8 | 1 (0.09) | 18 (0.07) |  | 5.26 % |
| 9 | 2 (0.18) | 12 (0.04) |  | 14.29 % |
| 10 | 0 (0) | 2 (0.01) |  | 0 % |
| 14 | 0 (0) | 1 (0) |  | 0 % |
| 16 | 0 (0) | 1 (0) |  | 0 % |
| Unspecified | 0 (0) | 6 (0.02) |  | 0 % |
| Lesion Graft 19 Target Vessel* |  |  |  |  |
| Unspecified | 0 (0) | 1 (0) | NE | 0 % |
| Lesion Graft 20 Target Vessel* |  |  |  |  |
| 1 | 1 (0.09) | 19 (0.07) | <0.001 | 5 % |
| 2 | 0 (0) | 12 (0.04) |  | 0 % |
| 3 | 1 (0.09) | 19 (0.07) |  | 5 % |
| 4 | 11 (1.00) | 116 (0.43) |  | 8.66 % |
| 5 | 0 (0) | 5 (0.02) |  | 0 % |
| 7 | 1 (0.09) | 39 (0.14) |  | 2.5 % |
| 8 | 3 (0.27) | 18 (0.07) |  | 14.29 % |
| 9 | 1 (0.09) | 6 (0.02) |  | 14.29 % |
| 10 | 8 (0.73) | 52 (0.19) |  | 13.33 % |
| 11 | 0 (0) | 2 (0.01) |  | 0 % |
| 13 | 2 (0.18) | 20 (0.07) |  | 9.09 % |
| 14 | 0 (0) | 11 (0.04) |  | 0 % |
| 15 | 14 (1.28) | 153 (0.57) |  | 8.38 % |
| 16 | 1 (0.09) | 20 (0.07) |  | 4.76 % |
| 17 | 1 (0.09) | 2 (0.01) |  | 33.33 % |
| 20 | 0 (0) | 1 (0) |  | 0 % |
| Unspecified | 2 (0.18) | 25 (0.09) |  | 7.41 % |
| Lesion Graft 21 Target Vessel* |  |  |  |  |
| 1 | 0 (0) | 3 (0.01) | 0.269 | 0 % |
| 3 | 0 (0) | 1 (0) |  | 0 % |
| 4 | 2 (0.18) | 9 (0.03) |  | 18.18 % |
| 7 | 0 (0) | 5 (0.02) |  | 0 % |
| 9 | 0 (0) | 1 (0) |  | 0 % |
| 10 | 1 (0.09) | 4 (0.01) |  | 20 % |
| 13 | 0 (0) | 1 (0) |  | 0 % |
| 14 | 0 (0) | 3 (0.01) |  | 0 % |
| 15 | 0 (0) | 19 (0.07) |  | 0 % |
| 16 | 0 (0) | 3 (0.01) |  | 0 % |
| Unspecified | 0 (0) | 1 (0) |  | 0 % |
| Lesion Graft 22 Target Vessel* |  |  |  |  |
| 3 | 0 (0) | 1 (0) | 0.440 | 0 % |
| 4 | 1 (0.09) | 5 (0.02) |  | 16.67 % |
| 7 | 0 (0) | 1 (0) |  | 0 % |
| 8 | 0 (0) | 1 (0) |  | 0 % |
| 15 | 0 (0) | 2 (0.01) |  | 0 % |
| 16 | 0 (0) | 1 (0) |  | 0 % |
| 17 | 0 (0) | 1 (0) |  | 0 % |
| Unspecified | 0 (0) | 2 (0.01) |  | 0 % |
| Lesion Graft 23 Target Vessel* |  |  |  |  |
| Unspecified | 0 (0) | 3 (0.01) | NE | 0 % |
| Lesion Graft 24 Target Vessel* | 0 (0) | 0 (0) | NE |  |
| Lesion Graft 25 Target Vessel* |  |  |  |  |
| Unspecified | 0 (0) | 1 (0) | NE | 0 % |
| Ostial | 135 (12.30) | 2379 (8.84) | <0.001 | 5.37 % |
| CTO >3 months | 98 (8.93) | 2570 (9.55) | 0.523 | 3.67 % |
| Calcified Lesion | 59 (5.37) | 725 (2.69) | <0.001 | 7.53 % |
| LMS | 54 (4.92) | 641 (2.38) | <0.001 | 7.77 % |
| Thrombus | 30 (2.73) | 790 (2.94) | 0.763 | 3.66 % |
| Bifurcation | 77 (7.01) | 2251 (8.37) | 0.125 | 3.31 % |
| Pre PCI % of Stenosis, mean (SD) | n=989 | n=24580 | 0.793 |  |
|  | 86.34 (25.47) | 86.19 (17.61) |  |  |
| Pre PCI TIMI Flow |  |  |  |  |
| TIMI-0 | 167 (15.21) | 4473 (16.62) | 0.248 | 3.6 % |
| TIMI-1 | 102 (9.29) | 2438 (9.06) |  | 4.02 % |
| TIMI-2 | 322 (29.33) | 7174 (26.66) |  | 4.3 % |
| TIMI-3 | 394 (35.88) | 9828 (36.52) |  | 3.85 % |
| Missing | 113 (10.29) | 2996 (11.13) |  | 3.63 % |
| Post PCI % of Stenosis, mean (SD) | n=991 | n=24153 | 0.020 |  |
|  | 5.51 (21.74) | 4.03 (19.52) |  |  |
| Post PCI TIMI Flow |  |  |  |  |
| TIMI-0 | 38 (3.46) | 731 (2.72) | 0.135 | 4.94 % |
| TIMI-1 | 7 (0.64) | 90 (0.33) |  | 7.22 % |
| TIMI-2 | 13 (1.18) | 254 (0.94) |  | 4.87 % |
| TIMI-3 | 981 (89.34) | 24241 (90.09) |  | 3.89 % |
| Missing | 59 (5.37) | 1593 (5.92) |  | 3.57 % |
| Estimated Lesion Length (mm), mean (SD) | n=999 | n=25011 | 0.098 |  |
|  | 28.37 (16.50) | 27.50 (16.29) |  |  |
| Perforation during PCI | 5 (0.46) | 76 (0.28) | 0.251 | 6.17 % |
| Lesion Result, successful | 1036 (94.35) | 25890 (96.21) | 0.002 | 3.85 % |
| Dissection during PCI |  |  |  |  |
| Flow limiting | 0 (0) | 22 (0.08) | 0.638 | 0 % |
| Non flow limiting | 9 (0.82) | 218 (0.81) |  | 3.96 % |
| No reflow, n (%) |  |  |  |  |
| Transient | 2 (0.18) | 83 (0.31) | 0.035 | 2.35 % |
| Persistent | 3 (0.27) | 13 (0.05) |  | 18.75 % |
| Maximum balloon size (mm), mean (SD) | n=1020 | n=25224 | 0.221 |  |
|  | 3.09 (0.54) | 3.13 (0.87) |  |  |
| Maximum balloon pressure (atm), mean (SD) | n=1003 | n=24811 | 0.004 |  |
|  | 16.52 (4.23) | 16.15 ( 4.00) |  |  |
| IVUS | 63 (5.74) | 1278 (4.75) | 0.152 | 4.7 % |
| OCT | 7 (0.64) | 236 (0.88) | 0.501 | 2.88 % |
| FFR | 8 (0.73) | 275 (1.02) | 0.424 | 2.83 % |
| Aspiration Catheter | 45 (4.10) | 1060 (3.94) | 0.852 | 4.07 % |
| POBA | 23 (2.09) | 373 (1.39) | 0.069 | 5.81 % |
| Angiojet | 0 (0) | 1 (0) | >0.999 | 0 % |
| Micro Catheter | 53 (4.83) | 1335 (4.96) | 0.897 | 3.82 % |
| Coil | 0 (0) | 0 (0) | NE |  |
| Rotablator | 38 (3.46) | 355 (1.32) | <0.001 | 9.67 % |
| Cutting/Scoring Balloon | 56 (5.10) | 899 (3.34) | 0.002 | 5.86 % |
| Mother-in-Child Catheter | 1 (0.09) | 32 (0.12) | >0.999 | 3.03 % |
| Embolic Protection |  |  |  |  |
| Filter | 4 (0.36) | 66 (0.25) | 0.471 | 5.71 % |
| Proximal | 0 (0) | 1 (0) |  | 0 % |
| Balloon | 0 (0) | 4 (0.01) |  | 0 % |
| Direct Stenting | 53 (4.83) | 1447 (5.38) | 0.509 | 3.53 % |
| Data were presented as n (%) unless otherwise stated. *Lesion graft number and anatomosis site number is based on the Modified AHA Coronary Segment Classification used in SYNTAX scoring (Ref: Yadav M, et al. Prediction of coronary risk by SYNTAX and derived scores: synergy between percutaneous coronary intervention with taxus and cardiac surgery. J Am Coll Cardiol. 2013;62(14):1219-1230) CCS, Canadian Cardiovascular Score; NSTEMI, Non-ST-elevation myocardial infarction; NYHA, New York Heart Association functional classification; PCI, percutaneous coronary intervention; SD, standard deviation; STEMI, ST-elevation myocardial infarction; UA, unstable angina; ACE, Angiotensin Converting Enzyme; ARB, Angiotensin Receptor Blocker; BMI, Body Mass Index; CAD, Coronary Artery Disease; CABG, Coronary Artery Bypass Grafting; CK/CKMB, creatine kinase/creatine kinase myocardial band; CSS, Canadian Cardiovascular Score; CT, Computerized tomography; CTO, Chronic Total Occlusion; FFR, Fractional Flow Reserve; IVUS, Intravascular Ultrasound; ; LBBB, Left Bundle Branch Block; LCx, Left Circumflex; LDL, low-density lipoproteins; LMS, Left Main Stem; LMWH, Low-Molecular-Weight Heparin; MDRD, Modification of Diet in Renal Disease; NYHA, New York Heart Association functional classification; OCT, optical coherence tomography; OHA, Oral Hyperglycemic Agents; PCI, percutaneous coronary intervention; POBA, Plain Old Balloon Angioplasty; RCA, Right Coronary Artery; TIA, Transient Ischemic Attack; TIMI, Thrombolysis in Myocardial Infarction; URL, Upper Reference Limit; VF, Ventricular Fibrillation; VT, Ventricular Fibrillation | | | | |

Supplementary Table 7. Patient demographics and disease characteristic by target vessel revascularization event

|  | **Target vessel revascularization** | |  |  |
| --- | --- | --- | --- | --- |
|  | **Yes** | **No** |  |  |
|  | **N=2654** | **N=25353** | **p-values** | **Target vessel revascularization rate** |
| Patient demographics | | | | |
| Age at admission, mean (SD) | 57.94 (9.938) | 58.18 (10.155) | 0.258 |  |
| Gender |  |  |  |  |
| Female | 398 (15.00) | 4458 (17.58) | 0.001 | 8.2 % |
| Male | 2256 (85.00) | 20895 (82.42) |  | 9.74 % |
| Ethnicity |  |  |  |  |
| Malay | 1530 (57.65) | 14469 (57.07) | <0.001 | 9.56 % |
| Chinese | 320 (12.06) | 3773 (14.88) |  | 7.82 % |
| Indian | 751 (28.30) | 6536 (25.78) |  | 10.31 % |
| Other Malaysian | 45 (1.70) | 436 (1.72) |  | 9.36 % |
| Foreigner | 8 (0.30) | 139 (0.55) |  | 5.44 % |
| Cardiac status at PCI procedure | | | | |
| Angina type |  |  |  |  |
| Atypical | 276 (10.40) | 3072 (12.12) | <0.001 | 8.24 % |
| Chronic Stable Angina | 1347 (50.75) | 12826 (50.59) |  | 9.5 % |
| UA | 421 (15.86) | 3087 (12.18) |  | 12 % |
| None | 558 (21.02) | 6014 (23.72) |  | 8.49 % |
| Missing | 52 (1.96) | 354 (1.40) |  | 12.81 % |
| CCS |  |  |  |  |
| Asymptomatic | 170 (6.41) | 2140 (8.44) | <0.001 | 7.36 % |
| CCS 1 | 820 (30.90) | 8432 (33.26) |  | 8.86 % |
| CCS 2 | 1208 (45.52) | 11537 (45.51) |  | 9.48 % |
| CCS 3 | 231 (8.70) | 1364 (5.38) |  | 14.48 % |
| CCS 4 | 72 (2.71) | 406 (1.60) |  | 15.06 % |
| Missing | 153 (5.76) | 1474 (5.81) |  | 9.4 % |
| NYHA |  |  |  |  |
| NYHA I | 1339 (50.45) | 14112 (55.66) | <0.001 | 8.67 % |
| NYHA II | 1006 (37.91) | 9042 (35.66) |  | 10.01 % |
| NYHA III | 156 (5.88) | 983 (3.88) |  | 13.7 % |
| NYHA IV | 25 (0.94) | 169 (0.67) |  | 12.89 % |
| Missing | 128 (4.82) | 1047 (4.13) |  | 10.89 % |
| Coronary Artery Disease |  |  |  |  |
| STEMI: Anterior | 205 (7.72) | 2291 (9.04) | <0.001 | 8.21 % |
| STEMI: Non anterior | 236 (8.89) | 1454 (5.74) |  | 13.96 % |
| STEMI: Unknown | 19 (0.72) | 194 (0.77) |  | 8.92 % |
| NSTEMI | 222 (8.36) | 1825 (7.20) |  | 10.85 % |
| UA | 104 (3.92) | 644 (2.54) |  | 13.9 % |
| Stable Ischemic heart disease | 1867 (70.35) | 18934 (74.68) |  | 8.98 % |
| Missing | 1 (0.04) | 11 (0.04) |  | 8.33 % |
| Clinical examination and baseline investigation | | | | |
| Height, cm, mean (SD) | n=2384 | n=22795 | 0.753 |  |
|  | 163.50 (8.22) | 163.44 (8.36) |  |  |
| Weight, kg, mean (SD) | n=2385 | n=22809 | 0.193 |  |
|  | 72.63 (13.60) | 72.25 (13.64) |  |  |
| BMI, kg/m^2^, mean (SD) | n=2368 | n=22686 | 0.200 |  |
|  | 27.14 (4.53) | 27.01 (4.53) |  |  |
| Heart rate at start of PCI, bpm, mean (SD) | n=2473 | n=23809 | 0.693 |  |
|  | 71.39 (15.33) | 71.53 (16.79) |  |  |
| Systolic blood pressure, mmHg, mean (SD) | n=2461 | n=23745 | 0.288 |  |
|  | 134.77 (25.46) | 135.33 (24.68) |  |  |
| Diastolic blood pressure, mmHg, mean (SD) | n=2460 | n=23723 | 0.675 |  |
|  | 76.52 (14.29) | 76.66 (16.36) |  |  |
| Baseline creatinine, mean (SD) | n=2601 | n=24920 | <0.001 |  |
|  | 129.85 (160.89) | 118.72 (120.91) |  |  |
| Total cholesterol, mean (SD) | n=2225 | n=21389 | 0.046 |  |
|  | 4.38 (1.15) | 4.33 (1.15) |  |  |
| LDL, mean (SD) | n=2199 | n=21133 | 0.085 |  |
|  | 2.49 (1.02) | 2.45 (1.13) |  |  |
| Sinus rhythm | 2450 (92.31) | 23532 (92.82) | 0.361 | 9.43 % |
| Atrial fibrillation | 31 (1.17) | 305 (1.20) | 0.949 | 9.23 % |
| Second and third atrioventricular block | 6 (0.23) | 34 (0.13) | 0.270 | 15 % |
| LBBB | 13 (0.49) | 73 (0.29) | 0.109 | 15.12 % |
| RBBB | 11 (0.41) | 123 (0.49) | 0.723 | 8.21 % |
| MDRD, mean (SD) | n=2601 | n=24920 | 0.184 |  |
|  | 73.58 (30.57) | 74.35 (27.97) |  |  |
| Cockcroft-Gault, mean (SD) | n=2354 | n=22566 | 0.935 |  |
|  | 77.50 (34.45) | 77.56 (33.34) |  |  |
| Non-Invasive Test |  |  |  |  |
| Stress exercise test | 64 (2.41) | 679 (2.68) | 0.585 | 8.61 % |
| Nuclear | 29 (1.09) | 342 (1.35) | 0.448 | 7.82 % |
| MRI | 22 (0.83) | 134 (0.53) | 0.129 | 14.1 % |
| Stress echo | 25 (0.94) | 224 (0.88) | 0.836 | 10.04 % |
| CT scan | 28 (1.06) | 503 (1.98) | 0.003 | 5.27 % |
| Functional Ischaemia |  |  |  |  |
| Positive | 307 (11.57) | 3171 (12.51) | 0.156 | 8.83 % |
| Negative | 19 (0.72) | 257 (1.01) |  | 6.88 % |
| Equivocal | 16 (0.60) | 118 (0.47) |  | 11.94 % |
| Not applicable | 1802 (67.90) | 16946 (66.84) |  | 9.61 % |
| Missing | 510 (19.22) | 4861 (19.17) |  | 9.5 % |
| Status before event for procedure at index date | | | | |
| Smoking status |  |  |  |  |
| Never | 954 (35.95) | 10231 (40.35) | <0.001 | 8.53 % |
| Former (quit >30 days) | 718 (27.05) | 5929 (23.39) |  | 10.8 % |
| Current (within last 30 days) | 612 (23.06) | 5612 (22.14) |  | 9.83 % |
| Missing | 370 (13.94) | 3581 (14.12) |  | 9.36 % |
| Dyslipidaemia | 1913 (72.08) | 18249 (71.98) | 0.943 | 9.49 % |
| Hypertension | 2036 (76.71) | 18838 (74.30) | 0.007 | 9.75 % |
| Diabetes |  |  |  |  |
| OHA | 1083 (40.81) | 9921 (39.13) |  | 9.84 % |
| Insulin | 438 (16.50) | 3446 (13.59) |  | 11.28 % |
| Non-pharmacology therapy diet therapy | 54 (2.03) | 587 (2.32) |  | 8.42 % |
| Unknown therapy | 85 (3.20) | 713 (2.81) |  | 10.65 % |
| Family history of premature cardiovascular disease | 413 (15.56) | 3747 (14.78) | 0.327 | 9.93 % |
| Myocardial infarction history | 1228 (46.27) | 12003 (47.34) | 0.316 | 9.28 % |
| Documented CAD | 1542 (58.10) | 14486 (57.14) | 0.316 | 9.62 % |
| New onset angina <2 weeks | 973 (36.66) | 8323 (32.83) | <0.001 | 10.47 % |
| History of heart failure | 111 (4.18) | 1033 (4.07) | 0.833 | 9.7 % |
| Cerebrovascular disease | 71 (2.68) | 640 (2.52) | 0.684 | 9.99 % |
| Peripheral vascular disease | 30 (1.13) | 215 (0.85) | 0.169 | 12.24 % |
| Chronic renal failure | 201 (7.57) | 1674 (6.60) | 0.062 | 10.72 % |
| Previous intervention | | | | |
| Previous PCI |  |  |  |  |
| < 365.25 days | 262 (9.87) | 2853 (11.25) | 0.056 | 8.41 % |
| ≥ 365.25 days | 300 (11.30) | 3006 (11.86) |  | 9.07 % |
| Previous CABG |  |  |  |  |
| < 365.25 days | 9 (0.34) | 57 (0.22) | 0.005 | 13.64 % |
| ≥ 365.25 days | 136 (5.12) | 994 (3.92) |  | 12.04 % |
| CATH lab characteristics | | | | |
| PCI status |  |  |  |  |
| Elective | 2292 (86.36) | 22908 (90.36) | <0.001 | 9.1 % |
| NSTEMI/UA | 146 (5.50) | 1041 (4.11) |  | 12.3 % |
| STEMI | 216 (8.14) | 1404 (5.54) |  | 13.33 % |
| Thrombolytic |  |  |  |  |
| <1 day | 27 (1.02) | 190 (0.75) | 0.184 | 12.44 % |
| ≥ 1 day | 29 (1.09) | 341 (1.35) |  | 7.84 % |
| Glycoprotein llb/llla Blockade |  |  |  |  |
| Prior | 26 (0.98) | 163 (0.64) | 0.002 | 13.76 % |
| During | 23 (0.87) | 131 (0.52) |  | 14.94 % |
| After | 8 (0.30) | 33 (0.13) |  | 19.51 % |
| Heparin | 2590 (97.59) | 24690 (97.38) | 0.613 | 9.49 % |
| LMWH | 77 (2.90) | 591 (2.33) | 0.078 | 11.53 % |
| Ticlopidine | 59 (2.22) | 571 (2.25) | 0.977 | 9.37 % |
| Fondaparinux | 179 (6.74) | 1282 (5.06) | 0.001 | 12.25 % |
| Bivalirudin | 0 (0) | 6 (0.02) | >0.999 | 0 % |
| Aspirin | 2577 (97.10) | 24626 (97.13) | 0.952 | 9.47 % |
| Clopidogrel | 2422 (91.26) | 23599 (93.08) | 0.001 | 9.31 % |
| 75mg | 1688 (63.60) | 16305 (64.31) | 0.012 | 9.38 % |
| 300mg | 636 (23.96) | 6363 (25.10) |  | 9.09 % |
| 600mg | 13 (0.49) | 126 (0.50) |  | 9.35 % |
| ≥1200mg | 0 (0) | 1 (0) |  | 0 % |
| Planned duration of clopidogrel ticlopidine |  |  |  |  |
| 1 month | 89 (3.35) | 1058 (4.17) | 0.450 | 7.76 % |
| 3 months | 94 (3.54) | 949 (3.74) |  | 9.01 % |
| 6 months | 122 (4.60) | 1391 (5.49) |  | 8.06 % |
| 12 months | 1883 (70.95) | 18934 (74.68) |  | 9.05 % |
| >12 months | 126 (4.75) | 1278 (5.04) |  | 8.97 % |
| Brachial | 11 (0.41) | 134 (0.53) | 0.517 | 7.59 % |
| Femoral | 1321 (49.77) | 11618 (45.82) | <0.001 | 10.21 % |
| Radial | 1415 (53.32) | 14169 (55.89) | 0.007 | 9.08 % |
| Closure device |  |  |  |  |
| Exoseal | 28 (1.06) | 235 (0.93) | <0.001 | 10.65 % |
| Seal | 70 (2.64) | 1167 (4.60) |  | 5.66 % |
| Suture | 12 (0.45) | 130 (0.51) |  | 8.45 % |
| Others | 14 (0.53) | 100 (0.39) |  | 12.28 % |
| LAD | 777 (29.28) | 7714 (30.43) | 0.229 | 9.15 % |
| LCx | 529 (19.93) | 4115 (16.23) | <0.001 | 11.39 % |
| RCA | 753 (28.37) | 5118 (20.19) | <0.001 | 12.83 % |
| Graft | 77 (2.90) | 535 (2.11) | 0.010 | 12.58 % |
| LMS | 64 (2.41) | 631 (2.49) | 0.858 | 9.21 % |
| Fluoroscopy time, mean (SD) | n=2406 | n=23019 | <0.001 |  |
|  | 25.14 (21.93) | 19.87 (23.47) |  |  |
| Contrast volume, mean (SD) | n=2432 | n=23417 | <0.001 |  |
|  | 198.22 (80.43) | 183.62 (66.47) |  |  |
| Procedure complications | | | | |
| Significant Periprocedural MI |  |  |  |  |
| Rise in CK/ CKMB > x3 URL | 3 (0.11) | 18 (0.07) |  | 14.29 % |
| Rise in Troponin > x5 URL | 2 (0.08) | 23 (0.09) |  | 8 % |
| ECG changes | 0 (0) | 5 (0.02) |  | 0 % |
| Unknown MI | 10 (0.38) | 43 (0.17) |  | 18.87 % |
| Emergency Reintervention/PCI |  |  |  |  |
| Stent thrombosis | 1 (0.04) | 19 (0.07) |  | 5 % |
| Dissection | 1 (0.04) | 5 (0.02) |  | 16.67 % |
| Cardiac perforation | 0 (0) | 0 (0) |  |  |
| Coronary perforation | 0 (0) | 0 (0) |  |  |
| New ischaemia | 0 (0) | 5 (0.02) |  | 0 % |
| Reinfarction | 0 (0) | 0 (0) |  |  |
| Cardiac tamponade | 0 (0) | 0 (0) |  |  |
| Unknown emergency | 1 (0.04) | 9 (0.04) |  | 10 % |
| Bail-out CABG | 0 (0) | 0 (0) | NE |  |
| Cardiogenic shock | 3 (0.11) | 22 (0.09) | 0.727 | 12 % |
| Arrhythmia (VT/VF/Brady) | 7 (0.26) | 65 (0.26) | >0.999 | 9.72 % |
| TIA/ Stroke | 0 (0) | 11 (0.04) | 0.615 | 0 % |
| Tamponade | 0 (0) | 7 (0.03) | >0.999 | 0 % |
| Contrast reaction | 3 (0.11) | 17 (0.07) | 0.430 | 15 % |
| New onset worsened heart failure | 2 (0.08) | 10 (0.04) | 0.317 | 16.67 % |
| Worsening renal impairment | 6 (0.23) | 50 (0.20) | 0.929 | 10.71 % |
| Bleeding |  |  |  |  |
| Minimal (Non-CNS bleeding, non-overt bleeding, <3g/dL Hb drop) | 7 (0.26) | 40 (0.16) | 0.427 | 14.89 % |
| Minor (Non-CNS bleeding with 3-5g/dL Hb drop) | 1 (0.04) | 17 (0.07) |  | 5.56 % |
| Major (Any intracranial bleed or other bleeding ≥ 5g/dL Hb drop) | 1 (0.04) | 4 (0.02) |  | 20 % |
| Unspecified bleeding | 0 (0) | 2 (0.01) |  | 0 % |
| Bleeding site |  |  |  |  |
| Retroperitoneal | 0 (0) | 2 (0.01) | 0.551 | 0 % |
| Percutaneous entry site | 6 (0.23) | 43 (0.17) |  | 12.24 % |
| Others | 1 (0.04) | 6 (0.02) |  | 14.29 % |
| Unspecified bleeding site | 2 (0.08) | 12 (0.05) |  | 14.29 % |
| Access site occlusion | 0 (0) | 9 (0.04) | >0.999 | 0 % |
| Loss of radial pulse | 0 (0) | 0 (0) | NE |  |
| Dissection | 4 (0.15) | 19 (0.07) | 0.269 | 17.39 % |
| Pseudo aneurysm |  |  |  |  |
| Ultrasound compression | 1 (0.04) | 6 (0.02) | 0.164 | 14.29 % |
| Surgery | 0 (0) | 1 (0.00) |  | 0 % |
| Others | 2 (0.08) | 8 (0.03) |  | 20 % |
| Unspecified pseudoaneurysm | 2 (0.08) | 5 (0.02) |  | 28.57 % |
| Medication prescribed post-PCI | | | | |
| Aspirin | 2521 (94.99) | 24249 (95.65) | 0.111 | 9.42 % |
| Clopidogrel | 2286 (86.13) | 22720 (89.61) | <0.001 | 9.14 % |
| Ticlopidine | 84 (3.17) | 690 (2.72) | 0.205 | 10.85 % |
| Statin | 2475 (93.26) | 23811 (93.92) | 0.160 | 9.42 % |
| Beta blocker | 1891 (71.25) | 17851 (70.41) | 0.406 | 9.58 % |
| ACE Inhibitor | 1408 (53.05) | 13061 (51.52) | 0.143 | 9.73 % |
| ARB | 383 (14.43) | 4142 (16.34) | 0.012 | 8.46 % |
| Warfarin | 23 (0.87) | 320 (1.26) | 0.095 | 6.71 % |
| PCI Procedure details | | | | |
| Coronary Lesion |  |  |  |  |
| De novo | 2436 (91.79) | 23656 (93.31) | 0.121 | 9.34 % |
| Restenosis (No prior stent) | 1 (0.04) | 36 (0.14) |  | 2.7 % |
| Stent thrombosis: Acute | 4 (0.15) | 23 (0.09) |  | 14.81 % |
| Stent thrombosis: Sub acute | 2 (0.08) | 22 (0.09) |  | 8.33 % |
| Stent thrombosis: Late | 1 (0.04) | 17 (0.07) |  | 5.56 % |
| Stent thrombosis: Very late | 0 (0) | 2 (0.01) |  | 0 % |
| In stent restenosis: DES | 93 (3.50) | 644 (2.54) |  | 12.62 % |
| In stent restenosis: BMS | 32 (1.21) | 310 (1.22) |  | 9.36 % |
| Others | 7 (0.26) | 55 (0.22) |  | 11.29 % |
| Missing | 78 (2.94) | 588 (2.32) |  | 11.71 % |
| Lesion Type |  |  |  |  |
| A | 68 (2.56) | 1115 (4.40) | <0.001 | 5.75 % |
| B1 | 394 (14.85) | 5454 (21.51) |  | 6.74 % |
| B2 | 460 (17.33) | 4859 (19.17) |  | 8.65 % |
| C | 1713 (64.54) | 13795 (54.41) |  | 11.05 % |
| Missing | 19 (0.72) | 130 (0.51) |  | 12.75 % |
| LMS lesion | 34 (1.28) | 415 (1.64) | 0.191 | 7.57 % |
| Lesion Graft 18 Target Vessel* |  |  |  |  |
| 2 | 0 (0) | 2 (0.01) | 0.680 | 0 % |
| 6 | 0 (0) | 1 (0) |  | 0 % |
| 7 | 6 (0.23) | 35 (0.14) |  | 14.63 % |
| 8 | 2 (0.08) | 17 (0.07) |  | 10.53 % |
| 9 | 0 (0) | 14 (0.06) |  | 0 % |
| 10 | 0 (0) | 2 (0.01) |  | 0 % |
| 14 | 0 (0) | 1 (0) |  | 0 % |
| 16 | 0 (0) | 1 (0) |  | 0 % |
| Unspecified | 1 (0.04) | 5 (0.02) |  | 16.67 % |
| Lesion Graft 19 Target Vessel* |  |  |  |  |
| Unspecified | 0 (0) | 1 (0) | NE | 0 % |
| Lesion Graft 20 Target Vessel* |  |  |  |  |
| 1 | 3 (0.11) | 17 (0.07) | 0.469 | 15 % |
| 2 | 1 (0.04) | 11 (0.04) |  | 8.33 % |
| 3 | 1 (0.04) | 19 (0.07) |  | 5 % |
| 4 | 17 (0.64) | 110 (0.43) |  | 13.39 % |
| 5 | 0 (0) | 5 (0.02) |  | 0 % |
| 7 | 6 (0.23) | 34 (0.13) |  | 15 % |
| 8 | 4 (0.15) | 17 (0.07) |  | 19.05 % |
| 9 | 0 (0) | 7 (0.03) |  | 0 % |
| 10 | 7 (0.26) | 53 (0.21) |  | 11.67 % |
| 11 | 0 (0) | 2 (0.01) |  | 0 % |
| 13 | 1 (0.04) | 21 (0.08) |  | 4.55 % |
| 14 | 2 (0.08) | 9 (0.04) |  | 18.18 % |
| 15 | 24 (0.90) | 143 (0.56) |  | 14.37 % |
| 16 | 1 (0.04) | 20 (0.08) |  | 4.76 % |
| 17 | 0 (0) | 3 (0.01) |  | 0 % |
| 20 | 0 (0) | 1 (0) |  | 0 % |
| Unspecified | 2 (0.08) | 25 (0.10) |  | 7.41 % |
| Lesion Graft 21 Target Vessel* |  |  |  |  |
| 1 | 0 (0) | 3 (0.01) | 0.141 | 0 % |
| 3 | 0 (0) | 1 (0) |  | 0 % |
| 4 | 2 (0.08) | 9 (0.04) |  | 18.18 % |
| 7 | 3 (0.11) | 2 (0.01) |  | 60 % |
| 9 | 0 (0) | 1 (0) |  | 0 % |
| 10 | 1 (0.04) | 4 (0.02) |  | 20 % |
| 13 | 0 (0) | 1 (0) |  | 0 % |
| 14 | 0 (0) | 3 (0.01) |  | 0 % |
| 15 | 1 (0.04) | 18 (0.07) |  | 5.26 % |
| 16 | 0 (0) | 3 (0.01) |  | 0 % |
| Unspecified | 0 (0) | 1 (0) |  | 0 % |
| Lesion Graft 22 Target Vessel* |  |  |  |  |
| 3 | 1 (0.04) | 0 (0) | 0.322 | 100 % |
| 4 | 1 (0.04) | 5 (0.02) |  | 16.67 % |
| 7 | 0 (0) | 1 (0) |  | 0 % |
| 8 | 0 (0) | 1 (0) |  | 0 % |
| 15 | 0 (0) | 2 (0.01) |  | 0 % |
| 16 | 0 (0) | 1 (0) |  | 0 % |
| 17 | 0 (0) | 1 (0) |  | 0 % |
| Unspecified | 0 (0) | 2 (0.01) |  | 0 % |
| Lesion Graft 23 Target Vessel* |  |  |  |  |
| Unspecified | 0 (0) | 3 (0.01) | NE | 0 % |
| Lesion Graft 24 Target Vessel* | 0 (0) | 0 (0) | NE |  |
| Lesion Graft 25 Target Vessel* |  |  |  |  |
| Unspecified | 1 (0.04) | 0 (0) | NE | 100 % |
| Ostial | 244 (9.19) | 2270 (8.95) | 0.707 | 9.71 % |
| CTO >3 months | 445 (16.77) | 2223 (8.77) | <0.001 | 16.68 % |
| Calcified Lesion | 83 (3.13) | 701 (2.76) | 0.310 | 10.59 % |
| LMS | 64 (2.41) | 631 (2.49) | 0.858 | 9.21 % |
| Thrombus | 102 (3.84) | 718 (2.83) | 0.004 | 12.44 % |
| Bifurcation | 190 (7.16) | 2138 (8.43) | 0.026 | 8.16 % |
| Pre PCI % of Stenosis, mean (SD) | 2455 | 23114 | <0.001 |  |
|  | 89.38 (16.650) | 85.86 (18.079) |  |  |
| Pre PCI TIMI Flow |  |  |  |  |
| TIMI-0 | 715 (26.94) | 3925 (15.48) | <0.001 | 15.41 % |
| TIMI-1 | 278 (10.47) | 2262 (8.92) |  | 10.94 % |
| TIMI-2 | 665 (25.06) | 6831 (26.94) |  | 8.87 % |
| TIMI-3 | 743 (28.00) | 9479 (37.39) |  | 7.27 % |
| Missing | 253 (9.53) | 2856 (11.26) |  | 8.14 % |
| Post PCI % of Stenosis, mean (SD) | 2375 | 22769 | <0.001 |  |
|  | 10.56 (29.950) | 3.41 (18.066) |  |  |
| Post PCI TIMI Flow |  |  |  |  |
| TIMI-0 | 198 (7.46) | 571 (2.25) | <0.001 | 25.75 % |
| TIMI-1 | 20 (0.75) | 77 (0.30) |  | 20.62 % |
| TIMI-2 | 39 (1.47) | 228 (0.90) |  | 14.61 % |
| TIMI-3 | 2238 (84.33) | 22984 (90.66) |  | 8.87 % |
| Missing | 159 (5.99) | 1493 (5.89) |  | 9.62 % |
| Estimated Lesion Length (mm), mean (SD) | 2351 | 23659 | <0.001 |  |
|  | 31.08 (18.619) | 27.18 (16.006) |  |  |
| Perforation during PCI | 12 (0.45) | 69 (0.27) | 0.146 | 14.81 % |
| Lesion Result, successful | 2379 (89.64) | 24547 (96.82) | <0.001 | 8.84 % |
| Dissection during PCI |  |  |  |  |
| Flow limiting | 3 (0.11) | 19 (0.07) | 0.169 | 13.64 % |
| Non flow limiting | 29 (1.09) | 198 (0.78) |  | 12.78 % |
| No reflow, n (%) |  |  |  |  |
| Transient | 7 (0.26) | 78 (0.31) | 0.851 | 8.24 % |
| Persistent | 2 (0.08) | 14 (0.06) |  | 12.5 % |
| Maximum balloon size (mm), mean (SD) | 2356 | 23888 | 0.404 |  |
|  | 3.11 (0.932) | 3.13 (0.847) |  |  |
| Maximum balloon pressure (atm), mean (SD) | 2287 | 23527 | <0.001 |  |
|  | 16.47 (4.160) | 16.13 (3.990) |  |  |
| IVUS | 144 (5.43) | 1197 (4.72) | 0.117 | 10.74 % |
| OCT | 20 (0.75) | 223 (0.88) | 0.578 | 8.23 % |
| FFR | 12 (0.45) | 271 (1.07) | 0.003 | 4.24 % |
| Aspiration Catheter | 147 (5.54) | 958 (3.78) | <0.001 | 13.3 % |
| POBA | 46 (1.73) | 350 (1.38) | 0.168 | 11.62 % |
| Angiojet | 0 (0) | 1 (0) | >0.999 | 0 % |
| Micro Catheter | 205 (7.72) | 1183 (4.67) | <0.001 | 14.77 % |
| Coil | 0 (0) | 0 (0) | NE |  |
| Rotablator | 52 (1.96) | 341 (1.35) | 0.013 | 13.23 % |
| Cutting/Scoring Balloon | 79 (2.98) | 876 (3.46) | 0.216 | 8.27 % |
| Mother-in-Child Catheter | 4 (0.15) | 29 (0.11) | 0.549 | 12.12 % |
| Embolic Protection |  |  |  |  |
| Filter | 9 (0.34) | 61 (0.24) | 0.565 | 12.86 % |
| Proximal | 0 (0) | 1 (0) |  | 0 % |
| Balloon | 0 (0) | 4 (0.02) |  | 0 % |
| Direct Stenting | 75 (2.83) | 1425 (5.62) | <0.001 | 5 % |
| Data were presented as n (%) unless otherwise stated. *Lesion graft number and anatomosis site number is based on the Modified AHA Coronary Segment Classification used in SYNTAX scoring (Ref: Yadav M, et al. Prediction of coronary risk by SYNTAX and derived scores: synergy between percutaneous coronary intervention with taxus and cardiac surgery. J Am Coll Cardiol. 2013;62(14):1219-1230) CCS, Canadian Cardiovascular Score; NSTEMI, Non-ST-elevation myocardial infarction; NYHA, New York Heart Association functional classification; PCI, percutaneous coronary intervention; SD, standard deviation; STEMI, ST-elevation myocardial infarction; UA, unstable angina; ACE, Angiotensin Converting Enzyme; ARB, Angiotensin Receptor Blocker; BMI, Body Mass Index; CAD, Coronary Artery Disease; CABG, Coronary Artery Bypass Grafting; CK/CKMB, creatine kinase/creatine kinase myocardial band; CSS, Canadian Cardiovascular Score; CT, Computerized tomography; CTO, Chronic Total Occlusion; FFR, Fractional Flow Reserve; IVUS, Intravascular Ultrasound; ; LBBB, Left Bundle Branch Block; LCx, Left Circumflex; LDL, low-density lipoproteins; LMS, Left Main Stem; LMWH, Low-Molecular-Weight Heparin; MDRD, Modification of Diet in Renal Disease; NYHA, New York Heart Association functional classification; OCT, optical coherence tomography; OHA, Oral Hyperglycemic Agents; PCI, percutaneous coronary intervention; POBA, Plain Old Balloon Angioplasty; RCA, Right Coronary Artery; TIA, Transient Ischemic Attack; TIMI, Thrombolysis in Myocardial Infarction; URL, Upper Reference Limit; VF, Ventricular Fibrillation; VT, Ventricular Fibrillation | | | | |
